# Spatiotemporal analysis of soluble aggregates and autophagy markers in the R6/2 mouse model

**DOI:** 10.1038/s41598-020-78850-w

**Published:** 2021-01-08

**Authors:** M. J. Vijay Kumar, Devanshi Shah, Mridhula Giridharan, Niraj Yadav, Ravi Manjithaya, James P. Clement

**Affiliations:** 1 Neuroscience Unit, Jawaharlal Nehru Centre for Advanced Scientific Research, Jakkur, Bangalore, India; 2 Molecular Biology and Genetics Unit, Jawaharlal Nehru Centre for Advanced Scientific Research, Jakkur, Bangalore, India

**Keywords:** Macroautophagy, Huntington's disease

## Abstract

Maintenance of cellular proteostasis is vital for post-mitotic cells like neurons to sustain normal physiological function and homeostasis, defects in which are established hallmarks of several age-related conditions like AD, PD, HD, and ALS. The Spatio-temporal accumulation of aggregated proteins in the form of inclusion bodies/plaques is one of the major characteristics of many neurodegenerative diseases, including Huntington’s disease (HD). Toxic accumulation of HUNTINGTIN (HTT) aggregates in neurons bring about the aberrant phenotypes of HD, including severe motor dysfunction, dementia, and cognitive impairment at the organismal level, in an age-dependent manner. In several cellular and animal models, aggrephagy induction has been shown to clear aggregate-prone proteins like HTT and ameliorate disease pathology by conferring neuroprotection. In this study, we used the mouse model of HD, R6/2, to understand the pathogenicity of mHTT aggregates, primarily focusing on autophagy dysfunction. We report that basal autophagy is not altered in R6/2 mice, whilst being functional at a steady-state level in neurons. Moreover, we tested the efficacy of a known autophagy modulator, Nilotinib (Tasigna™), presently in clinical trials for PD, and HD, in curbing mHTT aggregate growth and their potential clearance, which was ineffective in both inducing autophagy and rescuing the pathological phenotypes in R6/2 mice.

## Introduction

Dysfunctional proteostasis is a significant cause of many neurodegenerative diseases such as Alzheimer’s, Parkinson’s, and Huntington’s, where impairment in protein degradation pathways lead to the accumulation of toxic inter- and intracellular aggregates^1–3^. Macroautophagy (herein autophagy) is a major intracellular recycling pathway that contributes to maintaining cellular proteostasis by degrading misfolded proteins and aggregates^4^. Autophagy involves the de novo formation of double-membrane vesicles known as autophagosomes that capture cellular cytoplasmic contents, including superfluous or damaged organelles and proteins. These autophagosomes eventually fuse with lysosomes to form autolysosomes, resulting in degradation of cargo by the acidic lysosomal hydrolases^5^. The degradation of these contents results in the release of essential building blocks of biomolecules, such as amino acids and nucleotides that are recycled back to the cytosol. Thus, constant basal autophagy in cells contributes to maintaining cellular homeostasis. Aggrephagy is a selective form of autophagy, wherein the cargo is in the form of protein aggregates. It is a significant degradation pathway that functions to maintain proteostasis. Not surprisingly, several pieces of evidence support the importance of autophagy as a resistive mechanism in neurodegenerative diseases^4–8^.

Given that many neurodegenerative diseases are late-onset and age-related, even a minor alteration in protein homeostasis and defects in autophagic clearance of toxic intracellular aggregates may have additive effects that manifest the disease later in life^1^. This aggregate formation further compounded in post-mitotic cells where they cannot divide and dilute the aggregates, thus leading to its toxic build-up, thereby causing neuronal death. In neurodegenerative disorders, aggrephagy is impaired at almost every step, beginning with phagophore formation, maturation of autophagosomes, cargo loading and finally fusion with lysosomes for degradation^6,7,9,10^. Identification of many disease-associated genes, besides investigation into their functions in neurodegenerative diseases, also demonstrate strong correlations between these mutated genes and autophagy^4^. Also, the literature suggests that loss of autophagy function in the brain results in the development of neurodegenerative-like phenotypes. It has been shown that deletion of core autophagy genes such as *Atg5* and *Atg7* (ATG-AuTophaGy related) in brain-specific knockout mice models resulted in the accumulation of toxic ubiquitinated intra-neuronal aggregates and showed a neurodegenerative-like phenotype. These mice displayed a progressive loss of neurons and reduced motor coordination and lifespan^11,12^. However, overexpression of *Atg5* in mice resulted in increased basal autophagy, which correlated strongly with an extended lifespan, increased insulin sensitivity and improved motor function^13^. Interestingly, the neonatal lethality of systemic deletion of Atg5 is rescued even when it is re-expressed only in neurons^14^. The lifespan extension after autophagy induction has also been corroborated in a knock-in Beclin1 mouse model, where they show a clear increase in life span^15^. A recent report in C. elegans suggested that upregulation of a specific autophagy receptor, p62/SQSTM1, is sufficient to increase the lifespan and clear PolyQ aggregate^16^. Thus, upregulation of basal autophagy in these mouse models protects the neuronal cells from age-mediated death. Although extensive research has been carried out to understand the pathophysiology of such neurological disorders, targets of certain therapeutic drugs are not clear, leading to effective therapeutic intervention. One such intervention is to target pathways involved in regulating autophagy, which has been implicated as a promising strategy to identify scalable drug targets for many neurodegenerative diseases^4,5^. Detection and development of specific active modulators of autophagy are necessary and is highly beneficial for clinical interventions. As explained previously, autophagy is blocked at several steps in neurodegenerative conditions. Thus, several molecules have been identified that modulate autophagy at various steps^17–19^. Identification of novel small molecules of autophagy modulators has gained importance for the past 15 years, and many reports have shown that autophagy can be regulated at each step with the interventions of small molecules and has high therapeutic value for the treatment of many severe forms of diseases including neurodegenerative diseases^20^.

Huntington’s disease (HD) is an autosomal dominant trinucleotide repeat pathology caused by an unstable expansion of CAG repeats, the trinucleotide encoding the amino acid glutamine in the exon 1 of the *HUNTINGTIN* gene^21,22^. This extended polyglutamine tract in mutant HUNTINGTIN (mHTT) protein misfolds to form intra-nuclear and cytoplasmic aggregates, leading to neurodegeneration characterised by loss of efferent medium spiny neurons in the striatum of basal ganglia^23,24^. As such, the presence of toxic HTT aggregates in neurons is responsible for failures of several fundamental cellular processes characterised by severe motor dysfunction, dementia, and cognitive impairment^25–27^. Studies have shown that aggrephagy is a significant degradation pathway for mHTT and is impaired in HD^5,6^. Upregulating autophagy by small molecules enhance clearance of aggregate-prone intracytoplasmic mHTT protein, thereby ameliorating disease pathology and conferring neuroprotective roles in various cellular and animal models of HD^28^.

In this study, we have used a robust mouse model for HD, R6/2, to understand the proteostasis defects concerning pathological significance in autophagy dysfunction^29^. Despite being a widely used model to study HD, basal autophagy in R6/2 or any other related mouse models have not been characterised. Here, we have studied the developmental profile across different stages of disease progression by investigating the expression profile for ATG proteins and dynamics of mHTT formation, starting from 2 to 12 weeks in wild-type and R6/2 mice. Moreover, studies have shown that inhibition of BCR-ABL1 (Breakpoint Cluster Region—Abelson murine leukaemia) tyrosine kinase by Nilotinib (Tasigna™) protects neurons from dying against MPTP (1-methyl-4-phenyl-1,2,3,6-tetrahydropyridine) toxicity in a pre-clinical mouse model of Parkinson’s disease and enhances the clearance of α-SYNUCLEIN^30,31^. Interestingly, Nilotinib (Tasigna™) is in clinical trials for Parkinson’s disease and Huntington’s disease, but its potency has not been evaluated in any of the rodent models of neurodegenerative illnesses^32–35^. Therefore, we assessed the efficacy of Nilotinib (Tasigna™) (hereafter referred to as Tasigna) in ameliorating physiological and behavioural deficits, and to extend the lifespan of the R6/2 mouse. We have demonstrated in this study that basal autophagy was unaltered in R6/2 and administration of Tasigna in R6/2 did not induce any change in the expression of p62/SQSTM1, LC3B (Light chain 3B), and GABARAPL2 (GABA Type A Receptor-Associated Protein-Like 2). We further showed that Tasigna was ineffective in mHTT aggregate clearance and could not rescue the behavioural abnormalities in R6/2. We conclude that basal autophagy is unaltered in R6/2, with Tasigna being ineffective in the induction of autophagy, and subsequent rescue of behavioural deficits by mHTT aggregate clearance. Multiple efforts to rescue behavioural and pathophysiological characteristics of the model system in concern has not met with great success. As such, a combinatorial targeted therapy which can exhibit multiple effects in inducing autophagy flux might be beneficial in enhancing the clearance of toxic aggregates. Implementing and understanding the methodology above would aid in manoeuvring viable drugs as an effective therapeutic intervention in various neurodegenerative disorders.

## Results

### mHTT aggregates were detected from 2 weeks of age in R6/2

**Figure 1:**
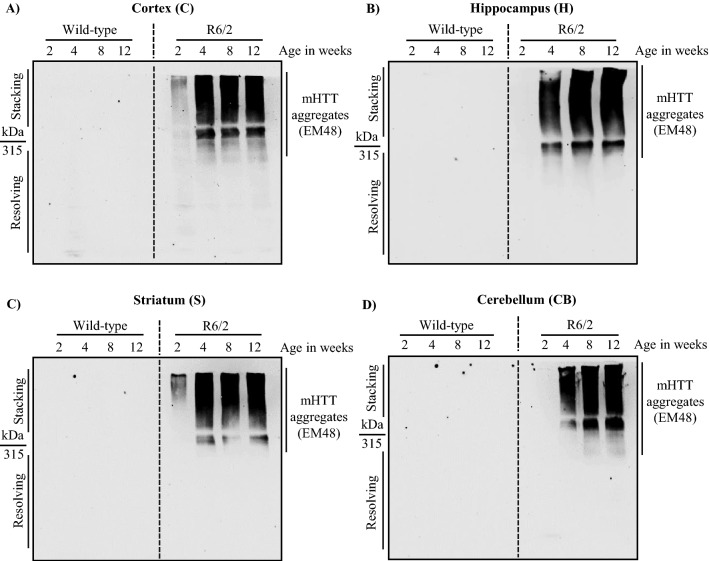
Dynamics of mHTT aggregate formation across different stages of disease progression in R6/2. (**A**)**–**(**D**) Representative blots for the expression of mHTT aggregates in R6/2 compared to wild-type control mice in the Cortex (C), Hippocampus (H), Striatum (S), and Cerebellum (CB) respectively at 2, 4, 8, and 12 weeks. mHTT aggregates were detected by EM48. N = 5 for 2 weeks, N = 7 for 4 and 8 weeks, and N = 12 for 12 weeks. N = number of mice. The genotypes are separated by vertical dashed lines. Image brightness and contrast were adjusted for representative purpose. Blots with different exposures and full uncropped raw blots are represented in the supplementary Figs. S1 and S7, respectively.

Studies have shown that mHTT aggregates are dynamic and exist in multiple conformations inside the cells leading to neuronal toxicity^24,36–38^. Immunohistochemical analysis has shown that UBIQUITIN positive HTT aggregates are present as early as PND 1 (Post Natal Day) in neostriatum and Hippocampus of R6/2 mice, and they increase in number and size as the disease progresses^39^. To check for the expression of mHTT aggregates, we performed immunoblot assay from different regions of the brain such as cortex, Hippocampus, striatum, and cerebellum (Fig. 1; Fig. S1). To identify the stage at which aggregates accumulate, we checked for the expression of mHTT aggregates across different stages of disease progression, i.e. 2, 4, 8, and 12 weeks of age. mHTT aggregates were first detected and started to accumulate from 2 weeks in cortex and striatum in R6/2 (Fig. 1A,C; Fig. S1(A-D), (I-L), whereas in Hippocampus and cerebellum, mHTT aggregates were detected from 4th week (Fig. 1B,D; Fig. S1 (E–H), (M–P). These results suggest that mHTT aggregates progressively accumulates across different regions of the brain in R6/2, contributing to rapid disease progression leading to neuronal death in concurrence with recent studies^40,41^.

### Ubiquitination is increased in the striatum at the end stage of disease progression in R6/2

Since mHTT aggregates were detected at early stages of disease progression, we checked for any alteration in the global ubiquitination profiles in different brain regions of the brain. Considering the fact that mHTT aggregates are of high molecular weight, we observed the presence of aggregates only in the stacking gel in western blot analysis (Fig. 1). In view of this, to identify the detectable changes, we assayed the ubiquitination profiles in both stacking and resolving gels from different regions of the brain across various stages of disease progression (Fig. S2). There was a significant increase in the ubiquitination pattern in both stacking and resolving in the region striatum in R6/2, compared to wild-type controls at 12 weeks of age. (Fig S2C. S2K, S2O). Interestingly, despite the presence of mHTT aggregates, there was no significant difference observed in the ubiquitination pattern in the other regions of the brain such as cortex (Fig. S2A, S2I, S2M), hippocampus (Fig. S2B, S2J, S2N), and cerebellum (Fig. S2D, S2L, S2P) across different stages of disease progression in R6/2. These results explain that due to the presence of mHTT aggregates, there is increased ubiquitination in the striatum, which is the most affected region of the brain in HD and also supports many studies suggesting the presence of intracellular ubiquitinated HUNTINGTIN aggregates in inclusion bodies^39,42–45^.

### Basal autophagy is unaltered in R6/2

**Figure 2:**
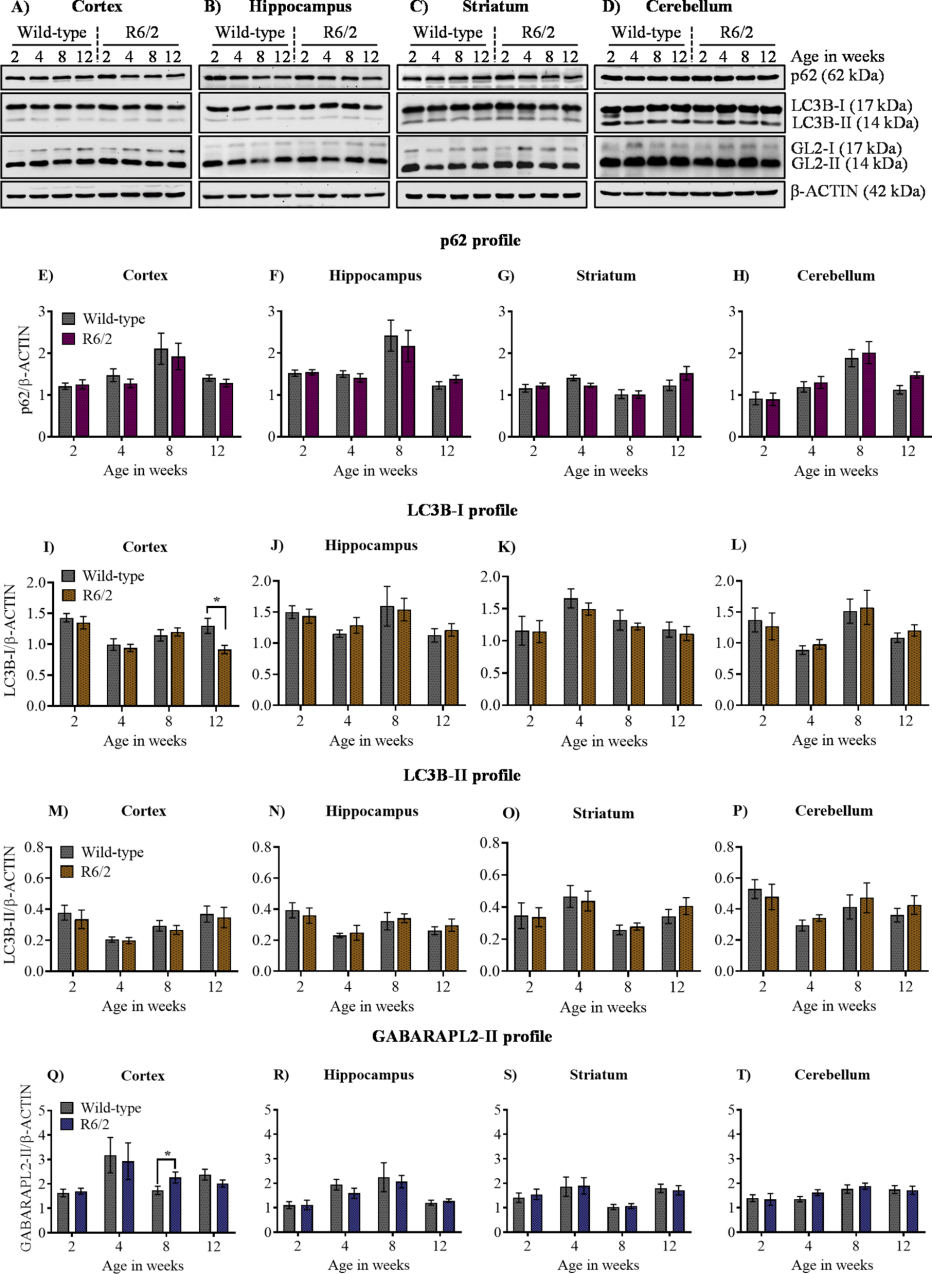
Basal autophagy is not altered across different stages of disease progression in R6/2. (**A**)–(**D**) Representative immunoblot for the expression of ATG proteins p62/SQSTM1, LC3B, GABARAPL2 (GL2) from 2, 4, 8, and 12 weeks in cortex, hippocampus, striatum and cerebellum, respectively. β-ACTIN is used as a loading control. (**E**)–(**H**) Pooled quantified bar graphs for p62 for cortex: Age x Genotype (F_(3,54)_ = 0.1322, p = 0.94), hippocampus: Age x Genotype (F_(3,54)_ = 0.4656, p = 0.70), striatum: Age x Genotype (F_(3,54)_ = 1.394, p = 0.25), and cerebellum: Age x Genotype (F_(3,54)_ = 0.6080, p = 0.61) respectively. (**I**)–(**L**) Pooled quantified bar graphs for LC3B-I for cortex: Age x Genotype (F_(3,54)_ = 2.380, p = 0.07); (12 weeks: *p < 0.05), hippocampus: Age x Genotype (F_(3,54)_ = 0.1890, p = 0.90), striatum: Age x Genotype (F_(3,54)_ = 0.09385, p = 0.96), and cerebellum: Age x Genotype (F_(3,54)_ = 0.1832, p = 0.90) respectively. (**M**)–(**P**) Quantified bar graphs for LC3B-II for cortex: Age x Genotype (F_(3,54)_ = 0.03193, p = 0.99), hippocampus: Age x Genotype (F_(3,54)_ = 0.2341, p = 0.87), striatum: Age x Genotype (F_(3,54)_ = 0.3311, p = 0.80), and cerebellum: Age x Genotype (F_(3,54)_ = 0.2769, p = 0.84) respectively. (**Q**)–(**T**) Summary of quantified bar graphs for GABARAPL2-II (GL2) for cortex: Age x Genotype (F_(3,54)_ = 0.5814, p = 0.62), hippocampus: Age x Genotype (F_(3,54)_ = 0.3486, p = 0.79), striatum: Age x Genotype (F_(3,54)_ = 0.07321, p = 0.97), and cerebellum: Age x Genotype (F_(3,54)_ = 0.3691, p = 0.77) respectively. N = 5 for 2 weeks, N = 7 for 4 and 8 weeks, and N = 12 for 12 weeks for both the genotypes wild-type, and R6/2. Error bars indicate ± SEM. (Mean and ± SEM values are represented in supplementary Tables 2A: Cortex, 2B: Hippocampus, 2C: Striatum, 2D: Cerebellum). N = number of mice. The genotypes are separated by vertical dashed lines. Statistical analysis was done by Two-way ANOVA followed by Bonferroni *post-hoc* test. Image brightness and contrast were adjusted for representative purpose. Full uncropped raw blots are represented in the supplementary Fig. S8.

Autophagy dysfunction plays a significant role in the pathophysiology of many neurodegenerative diseases^20^. Studies have shown that autophagy in the brain is tightly controlled and regulated^46,47^. To understand basal level autophagy regulation in the R6/2 mouse brain, we checked for the expression of key ATG proteins p62/SQSTM1, LC3B, and GABARAPL2 (referred henceforth as GL2) by immunoblotting from the cortex, Hippocampus, striatum, and cerebellum across different stages of disease progression as mentioned earlier. p62/SQSTM1, a UBIQUITIN-binding adaptor protein, binds to the cargo and drives its degradation by autophagy. There was no significant difference observed in the levels of p62/SQSTM1 in the cortex (Fig. 2A,E), Hippocampus (Fig. 2B,F), striatum (Fig. 2C,G), and cerebellum (Fig. 2D,H) in all the four age groups in R6/2 mice compared to wild-type control mice. LC3B and GABARAPL2 are considered as autophagosome markers, which labels the double membrane mature autophagosomes and exist in lipidated (phosphatidylethanolamine conjugated) as well as non-lipidated (unconjugated) forms, the former being the active one. There was no significant difference observed in the levels of LC3B-I and LC3B-II in the cortex (Fig. 2A,I,M), hippocampus (Fig. 2B,J,N), striatum (Fig. 2C,K,O), and cerebellum (Fig. 2D,L,P) in all the four age groups, except for 12 weeks, where there was a significant decrease in the expression of LC3B-I in cortex in R6/2. However, GABARAPL2-II showed no significant difference in the cortex (Fig. 2A,Q), Hippocampus (Fig. 2B,R), striatum (Fig. 2C,S), and cerebellum (Fig. 2D,T) in all four age groups in R6/2 mice, except for the increase in the expression at 8 weeks in the cortex. Despite the accumulation of toxic mHTT aggregates in these regions, as detected by mEM48 antibody, from 2 weeks, the basal level of autophagy was not altered in R6/2 during those development stages. In conclusion, western blot analysis showed that there were no significant changes observed in the expression of key ATG proteins p62/SQSTM1, LC3B-II, GABARAPL2-II in different regions of the brain across various stages of disease progression in R6/2 mice. The only significant difference found in levels of LC3B-I and GABARAPL2-II in 12 and 8 weeks in cortex respectively, might suggest an impairment in the formation and maturation of autophagosome. However, these results are inconclusive and need to be further validated by different ATG assays. Overall, our results further validate an earlier study in which the basal autophagy remained unaltered, but stringently regulated in the HD mouse model^48^.

### mHTT aggregates form intranuclear inclusions and co-localises with p62/SQSTM1 in R6/2

**Figure 3:**
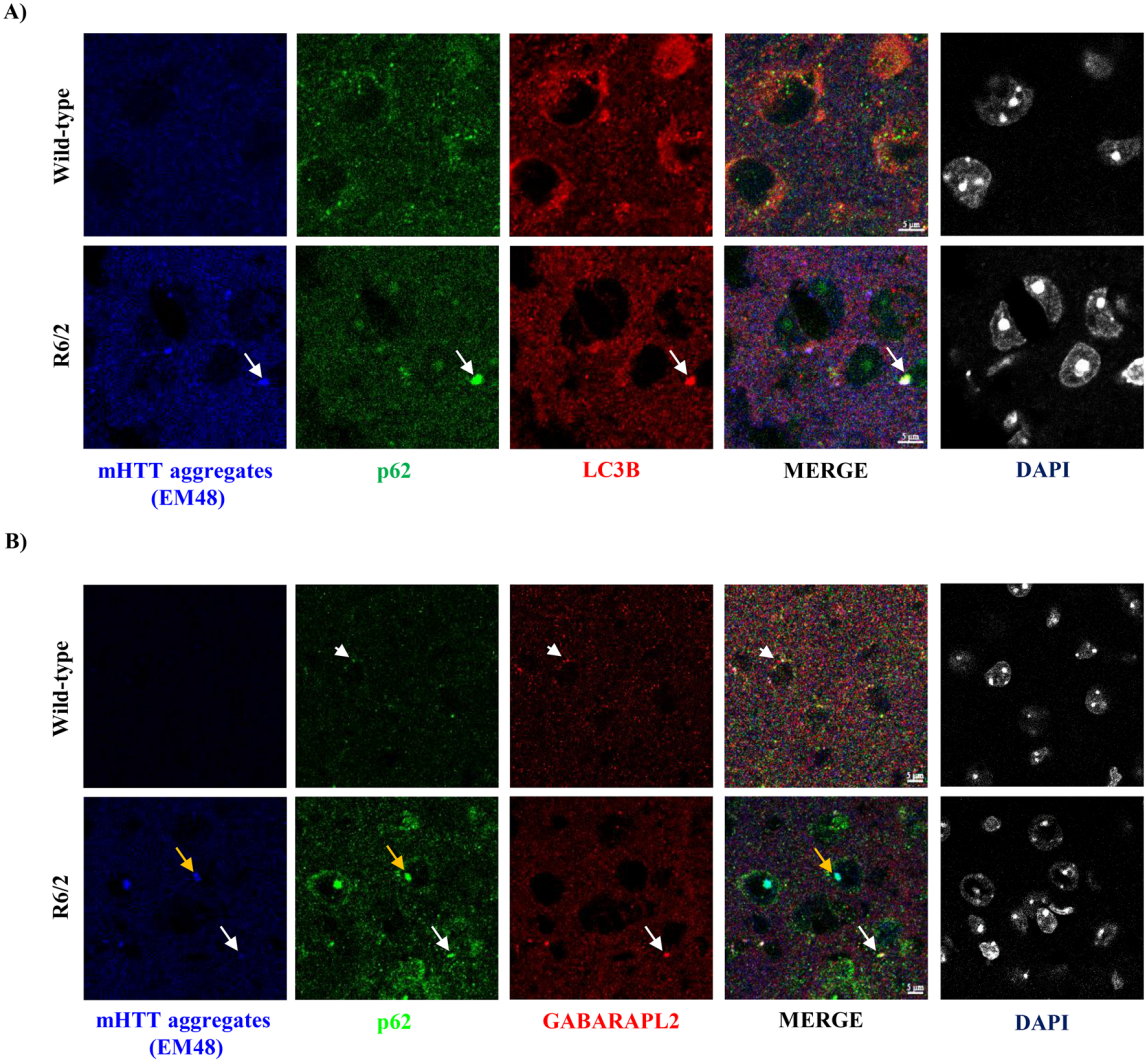
mHTT aggregates form intranuclear inclusions and get co-localised with p62/SQSTM1 in R6/2 in the striatum at 12 weeks. (**A**) Representative images for Immunohistochemistry for labelling of mHTT aggregates by EM48, and ATG proteins p62/SQSTM1, LC3B from wild-type and R6/2 mice. p62/SQSTM1 labelled in green, LC3B in red, and mHTT (EM48) in Blue. (**B**) Representative images for Immunohistochemistry for labelling of mHTT aggregates by EM48, and ATG proteins p62/SQSTM1, and GABARAPL2 from wild-type and R6/2 mice. p62/SQSTM1 labelled in green, GABARAPL2 in red, and mHTT (EM48) in Blue. Magnification = 63X. Scale bar = 5 μm. N = 3. White arrow indicates co-localisation of mHTT (EM48) aggregates with ATG markers. White arrowheads indicate co-localisation of p62/SQSTM1 with GABARAPL2. Yellow arrows indicate co-localisation of mHTT (EM48) aggregates with p62/SQSTM1. N = number of mice.

Having observed no change in basal autophagy in R6/2, we asked whether there is any change in the expression and co-localisation of key ATG-related proteins p62/SQSTM1, LC3B, GABARAPL2, and expression of mHTT aggregates by Immunohistochemistry at 12 weeks in the striatum of R6/2. We found that the mHTT aggregates formed intranuclear inclusions and co-localised with an adaptor protein, p62/SQSTM1, where it showed distinct punctate-like structures inside the nucleus in R6/2 but diffused pattern was observed in wild-type mice (Fig. 3A,B). There was no change in the expression pattern of LC3B and GABARAPL2 in R6/2 at 12 weeks in the striatum when compared to the wild-type counterparts. However, LC3B and GABARAPL2 did not wholly co-localise with p62/SQSTM1 and mHTT aggregates, indicating impairment in cargo loading leading to autophagy dysfunction in R6/2 (Fig. 3A,B).

### Nilotinib (Tasigna™) induces autophagy in HeLa cells

Autophagy is the process of sequestering the cargo into the double membrane autophagic vesicles and fusing them with lysosomes, thereby degrading the cytoplasmic contents. To validate the efficiency of Tasigna in inducing autophagy, HeLa cells were treated with varying concentrations of the drug for 2 h and observed that Tasigna induces autophagy in a dosage-dependent manner. Tasigna (1 mM) treatment induces autophagy when compared to controls, as revealed by the significant increase in the expression of LC3B-II (Fig S3A). Immunoblot results suggest that Tasigna enhanced the conversion of LC3B-I to LC3B-II compared to the control treatment in a dosage-dependent manner (Fig S3B). Treatment with bafilomycin A_1_ and 6BIO was used as a negative and positive modulator of autophagy, respectively. These results suggest that Nilotinib (Tasigna™) induces autophagy and as per the previous reports^49–51^.

### Nilotinib (Tasigna™) is not effective in improving the motor functions in R6/2

**Figure 4:**
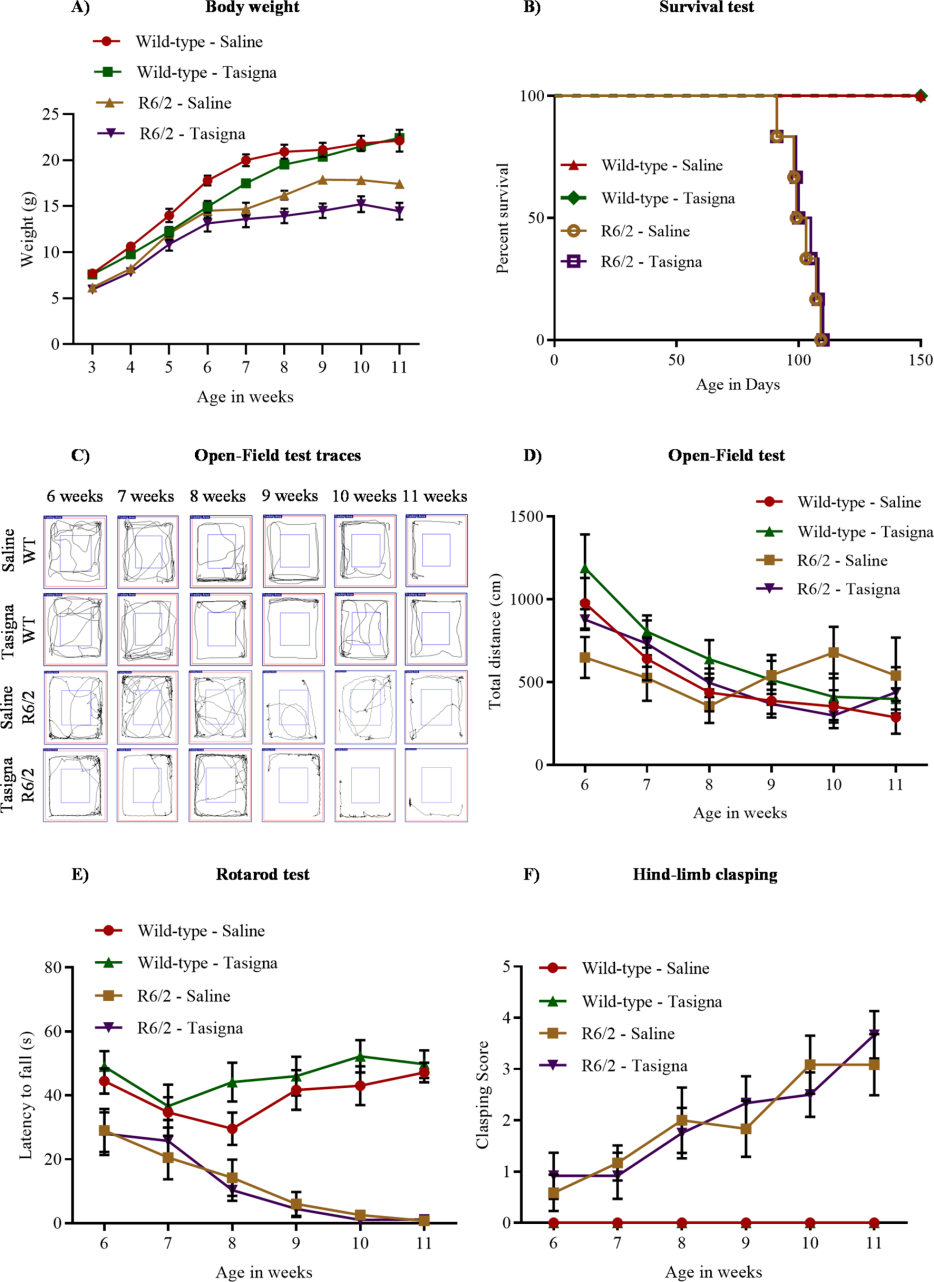
Nilotinib (Tasigna™) is ineffective in rescuing the motor functions and improving the survival rate in R6/2. (**A**) Line graph showing no effect of Tasigna on weight gain in R6/2, and wild-type control mice across different stages of disease progression. R6/2-Saline: N = 12 (M); R6/2-Tasigna: N = 12 (M); WT-Saline: N = 12 (M); WT-Tasigna: N = 12 (M); Age x Treatment x Genotype (F _(8, 8)_ = 2.079, p = 0.03). (**B**) Kaplan–Meier survival curve showing no increase in life span in Tasigna treated R6/2 mice compared to saline-treated mice (R6/2—Saline: N = 12 (M); R6/2-Tasigna: N = 12 (M)). (**C**) Representative traces showing no significant improvement in the distance travelled in Open-Field test in Tasigna treated group in R6/2 compared to the saline-treated group from 6 to 11 weeks. WT-Saline and WT-Tasigna groups also showing no significant difference in the distance travelled. (**D**) Line graph depicting total distance travelled in Open-field test from 6 to 11 weeks in Tasigna and saline-treated ones in both the genotypes. R6/2-Saline: N = 12 (M); R6/2-Tasigna: N = 12 (M); WT-Saline: N = 12 (M); WT-Tasigna: N = 12 (M); Age x Treatment x Genotype (F _(5, 5)_ = 0.560, p = 0.73). (**E**) Line graph showing no significant improvement in latency to fall in Rotarod test in R6/2 from 6 to 11 weeks in Tasigna-treated group compared to saline-treated. Tasigna does not affect WT control mice from 6 to 11 weeks. R6/2-Saline: N = 12 (M); R6/2-Tasigna: N = 12 (M); WT-Saline: N = 12 (M); WT-Tasigna: N = 12 (M); Age x Treatment x Genotype (F _(5, 5)_ = 0.5618, p = 0.72). (**F**) Line graph showing Tasigna having no significant effect in delaying the onset of hind-limb clasping symptom in R6/2 from 6 to 11 weeks. WT control mice were showing no effect on Tasigna treatment. Scores were plotted based on the Racine scale from 0–5, as explained in the methods section. R6/2-Saline: N = 12 (M); R6/2-Tasigna: N = 12 (M); WT-Saline: N = 12 (M); WT-Tasigna: N = 12 (M); Age x Treatment x Genotype (F _(5, 5)_ = 0.4634, p = 0.80). Error bars indicate ± SEM. (Mean and ± SEM values are represented in supplementary Tables 3A: Bodyweight, 3B: Open-Field test, 3C: Rotarod test, 3D: Hind-limb clasping test). N = number of mice, M = Males. Statistical analysis was done by 3-way ANOVA followed by Tukey *post-hoc* test.

Since Tasigna was shown to be neuroprotective in MPTP-based Parkinson’s model^31^, we checked for the efficacy of Tasigna in rescuing or delaying the onset of behavioural impairments in R6/2. Tasigna treatment neither affected body weight (Fig. 4A) nor survival rate of WT and R6/2 (Fig. 4B). We next asked whether administering Tasigna would alleviate motor dysfunction in R6/2 in the open-field test and rotarod. Still, We observed no improvement neither in locomotion and exploratory behaviour nor in latency time to fall respectively in R6/2 (Fig. 4C,D,E). These results confirm that Tasigna is not effective in rescuing behavioural impairments in R6/2 that led us to ask the question of whether disease progression can be delayed in R6/2. To address this, we performed a hind-limb clasping test and found no delay in disease progression in Tasigna treated group compared to saline-treated R6/2 animals (Fig. 4F). Therefore, Tasigna was ineffective in rescuing or delaying the onset of behavioural symptoms in R6/2, suggesting that the drug is not a potent therapeutic molecule in the R6/2 mouse model of HD.

### Nilotinib (Tasigna™) does not induce autophagy in R6/2 and Wild-type control mice

**Figure 5:**
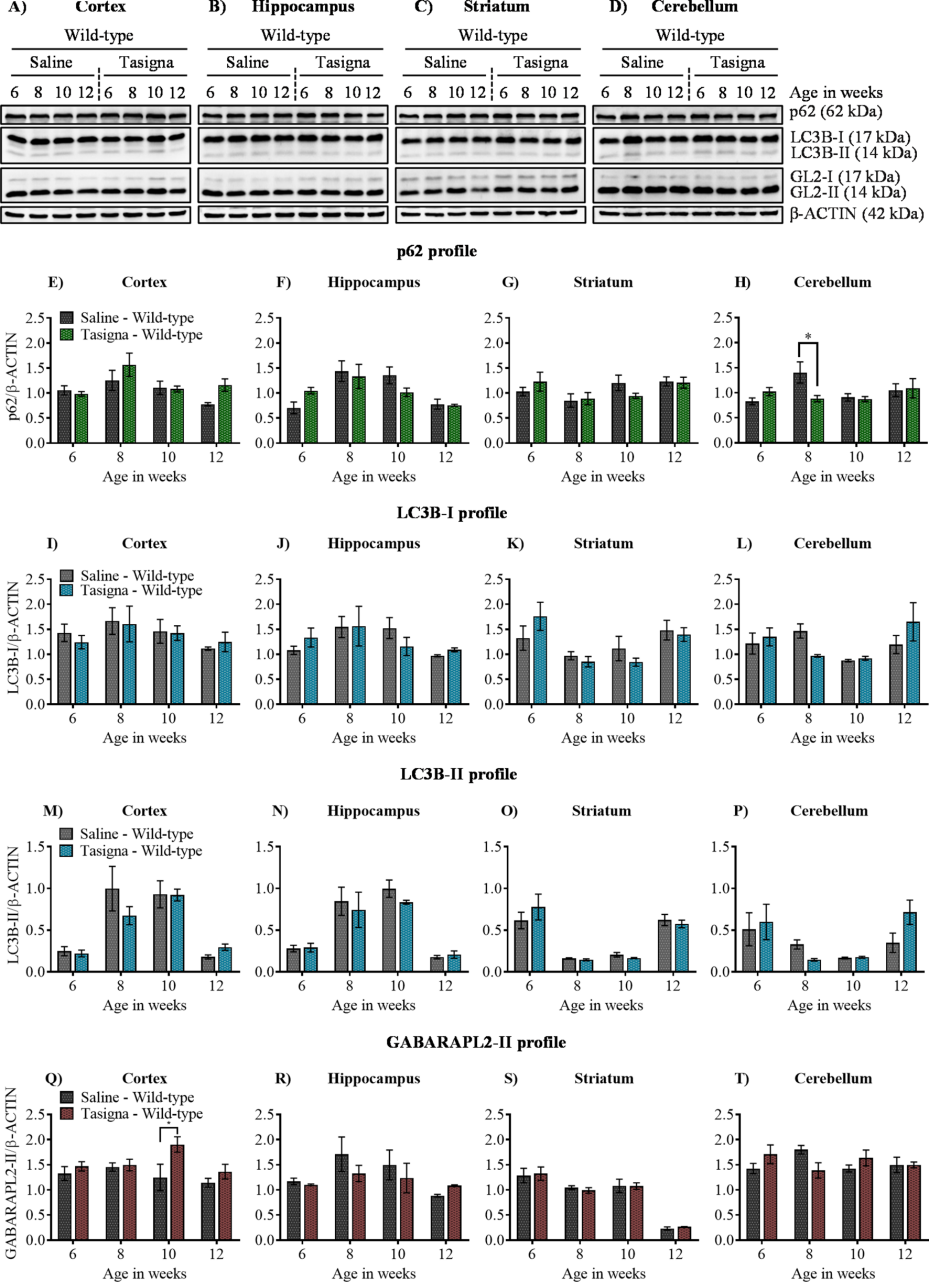
Nilotinib (Tasigna™) does not affect inducing autophagy in wild-type control mice. (**A**)–(**D**) Representative immunoblot for the expression of ATG proteins p62/SQSTM1, LC3B, GABARAPL2 (GL2) from 6, 8, 10 and 12 weeks in cortex, hippocampus, striatum and cerebellum, respectively. β-ACTIN is used as a loading control. (**E**)–(**H**) Pooled quantified bar graphs for p62/SQSTM1 for cortex: Age x treatment (F_(3,12)_ = 2.32, p = 0.12), hippocampus: Age x treatment (F_(3,12)_ = 1.87, p = 0.18), striatum: Age x treatment (F_(3,12)_ = 0.99, p = 0.42), and cerebellum: Age x treatment (F_(3,12)_ = 5.40, p = 0.01); (8 weeks: *p < 0.01) respectively. (**I**)–(**L**) Pooled quantified bar graphs for LC3B-I for cortex: Age x treatment (F_(3,12)_ = 0.59, p = 0.63), hippocampus: Age x treatment (F_(3,12)_ = 1.72, p = 0.21), striatum: Age x treatment (F_(3,12)_ = 1.13, p = 0.37), and cerebellum: Age x treatment (F_(3,12)_ = 3.06, p = 0.06) respectively. (**M**)–(**P**) Pooled quantified bar graphs for LC3B-II for cortex: Age x treatment (F_(3,12)_ = 1.65, p = 0.22), hippocampus: Age x treatment (F_(3,12)_ = 0.57, p = 0.64), striatum: Age x treatment (F_(3,12)_ = 1.22, p = 0.34), and cerebellum: Age x treatment (F_(3,12)_ = 2.22, p = 0.13) respectively. (**Q**)–(**T**) Pooled quantified bar graphs for GABARAPL2-II (GL2) for cortex: Age x treatment (F_(3,12)_ = 1.86, p = 0.18); (10 weeks: *p < 0.05), hippocampus: Age x treatment (F_(3,12)_ = 1.01, p = 0.42), striatum: Age x treatment (F_(3,12)_ = 0.08, p = 0.96), and cerebellum: Age x treatment (F_(3,12)_ = 3.74, p = 0.04) respectively. N = 3 (WT-Saline), and (WT-Tasigna) for all the 4 age groups 6, 8, 10, and 12 weeks. Error bars indicate ± SEM. (Mean and ± SEM values are represented in supplementary Tables 4A: Cortex, 4B: Hippocampus, 4C: Striatum, 4D: Cerebellum). N = number of mice. The treatment groups are separated by vertical dashed lines. Statistical analysis was done by Two-way ANOVA followed by Bonferroni *post-hoc* test. Image brightness and contrast were adjusted for representative purpose. Full uncropped raw blots are represented in the supplementary Fig. S10.

**Figure 6:**
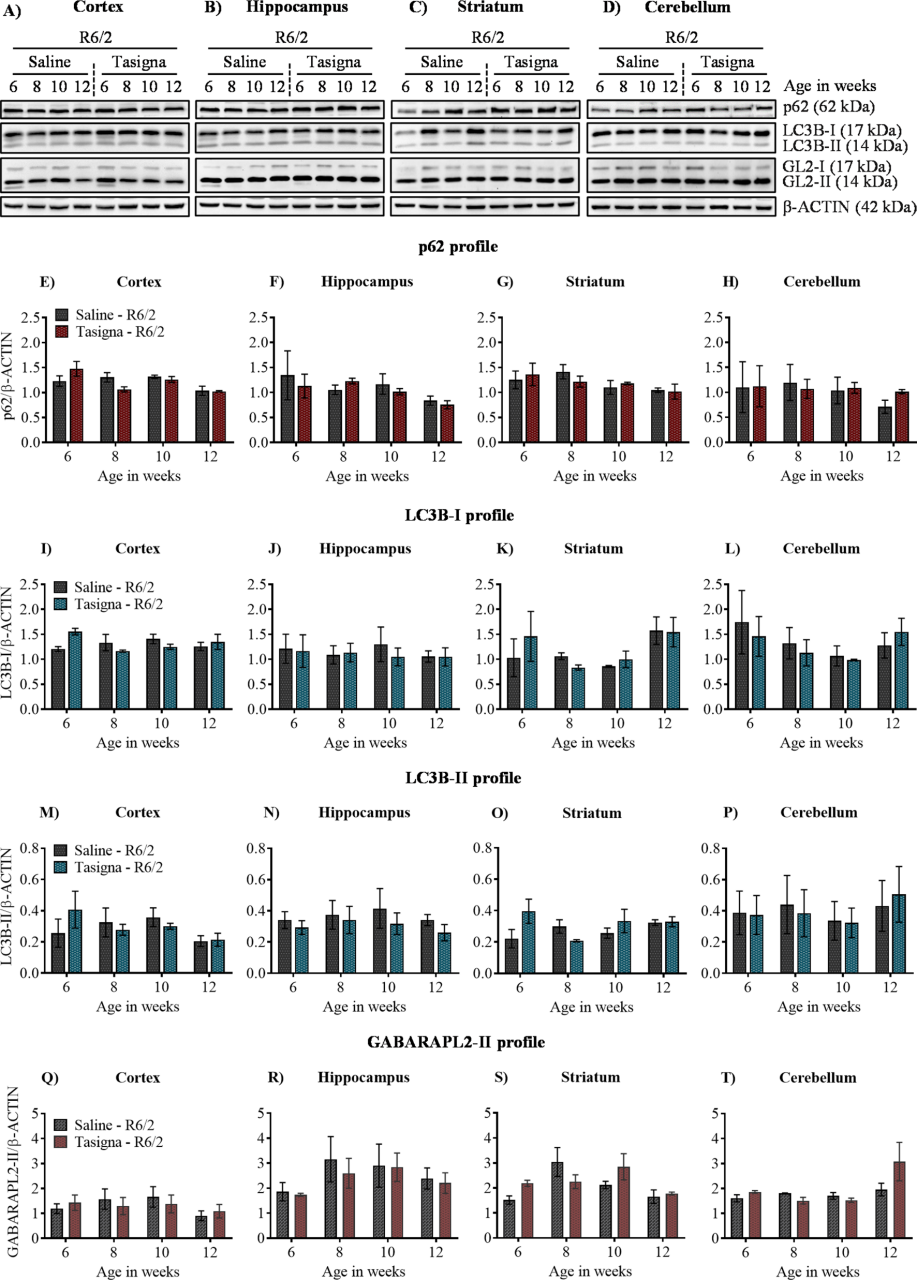
Nilotinib (Tasigna™) is ineffective in inducing autophagy across different stages of disease progression in R6/2. (**A**)–(**D**) Representative immunoblot for the expression of ATG proteins p62/SQSTM1, LC3B, GABARAPL2 (GL2) in cortex, hippocampus, striatum and cerebellum respectively from 6, 8, 10 and 12 weeks. β-ACTIN is used as a loading control. (**E**)–(**H**) Pooled quantified bar graphs for p62/SQSTM1 for cortex: Age x treatment (F_(3,12)_ = 2.29, p = 0.12), hippocampus: Age x treatment (F_(3,12)_ = 0.28, p = 0.83), striatum: Age x treatment (F_(3,12)_ = 0.39, p = 0.76), and cerebellum: Age x treatment (F_(3,12)_ = 0.27, p = 0.84) respectively. (**I**)–(**L**) Pooled quantified bar graphs for LC3B-I for cortex: Age x treatment (F_(3,12)_ = 3.27, p = 0.05), hippocampus: Age x treatment (F_(3,12)_ = 0.27, p = 0.84), striatum: Age x treatment (F_(3,12)_ = 0.85, p = 0.49), and cerebellum: Age x treatment (F_(3,12)_ = 0.55, p = 0.65) respectively. (**M**)–(**P**) Summary of quantified bar graphs for LC3B-II for cortex: Age x treatment (F_(3,12)_ = 0.91, p = 0.46), hippocampus: Age x treatment (F_(3,12)_ = 0.13, p = 0.93), striatum: Age x treatment (F_(3,12)_ = 4.68, p = 0.02), and cerebellum: Age x treatment (F_(3,12)_ = 0.43, p = 0.73) respectively. (**Q**)–(**T**) Pooled quantified bar graphs for GABARAPL2-II (GL2) for cortex: Age x treatment (F_(3,12)_ = 3.30, p = 0.05), hippocampus: Age x treatment (F_(3,12)_ = 0.20, p = 0.88), striatum: Age x treatment (F_(3,12)_ = 3.09, p = 0.06), and cerebellum: Age x treatment (F_(3,12)_ = 2.24, p = 0.13) respectively. N = 3 (R6/2-Saline), and (R6/2-Tasigna) for all the 4 age groups 6, 8, 10, and 12 weeks. Error bars indicate ± SEM. (Mean and ± SEM values are represented in supplementary Tables 5A: Cortex, 5B: Hippocampus, 5C: Striatum, 5D: Cerebellum). N = number of mice. The treatment groups are separated by vertical dashed lines. Statistical analysis was done by Two-way ANOVA followed by Bonferroni *post-hoc* test. Image brightness and contrast were adjusted for representative purpose. Full uncropped raw blots are represented in the supplementary Fig. S11.

Based on the above observations, we wondered whether Tasigna might induce autophagy in R6/2 mice, and yet the effect may not be reflected in the behaviour. Brain samples from different mice were prepared post-injection of Tasigna for immunoblotting and immunohistochemical analyses at various stages of disease progression viz. early HD (6 weeks of age in R6/2), progressive HD (8 and 10 weeks of age in R6/2), and end-stage HD (12 weeks of age in R6/2)^52,53^ from both R6/2 and wild-type control mice. Our initial studies on developmental profile have shown that expression of mHTT aggregates at 2 weeks was considerably low compared to the other age groups, and moreover, symptoms start appearing from 6 weeks in R6/2 mice. In this regard, we injected Tasigna at an early stage of disease progression, i.e. starting from 2 weeks to end-stage 12 weeks and checked for ATG protein expression and mHTT aggregate clearance from 6 weeks. We found that Tasigna had failed to induce autophagy in wild-type control mice, as there was no significant change observed in the expression of crucial ATG proteins, p62/SQSTM1, LC3B, and GABARAPL2 in all the four age groups in the cortex (Fig. 5A,E), Hippocampus (Fig. 5B,F), striatum (Fig. 5C,G), and cerebellum (Fig. 5D,H) respectively, except for p62/SQSTM1 in the cerebellum where there was a significant decrease in the expression level at 8 weeks. Moreover, there was no significant change observed in the expression of autophagosome markers LC3B-I and LC3B-II, and GABARAPL2 in all the four age groups in the cortex (Fig. 5A,I,M,Q), hippocampus (Fig. 5B,J,N,R), striatum (Fig. 5C,K,O,S), and cerebellum (Fig. 5D,L,P,T) respectively except for GABARAPL2 in the cortex where its expression significantly increased at 10 weeks. Tasigna did not affect autophagy induction in R6/2 mice as the expression of key ATG proteins remained unaltered in all the stages of disease progression, in the cortex (Fig. 6A,E,I,M,Q), Hippocampus (Fig. 6B,F,J,N,R), striatum (Fig. 6C,G,K,O,S), and cerebellum (Fig. 6D,H,L,P,T). Our results suggest that Tasigna did not affect the autophagy flux in both R6/2 and wild-type control mice across different stages of disease progression, which explains the lack of improvement in motor functions in R6/2.

### Nilotinib (Tasigna™) is unable to clear mHTT aggregates in R6/2

**Figure 7:**
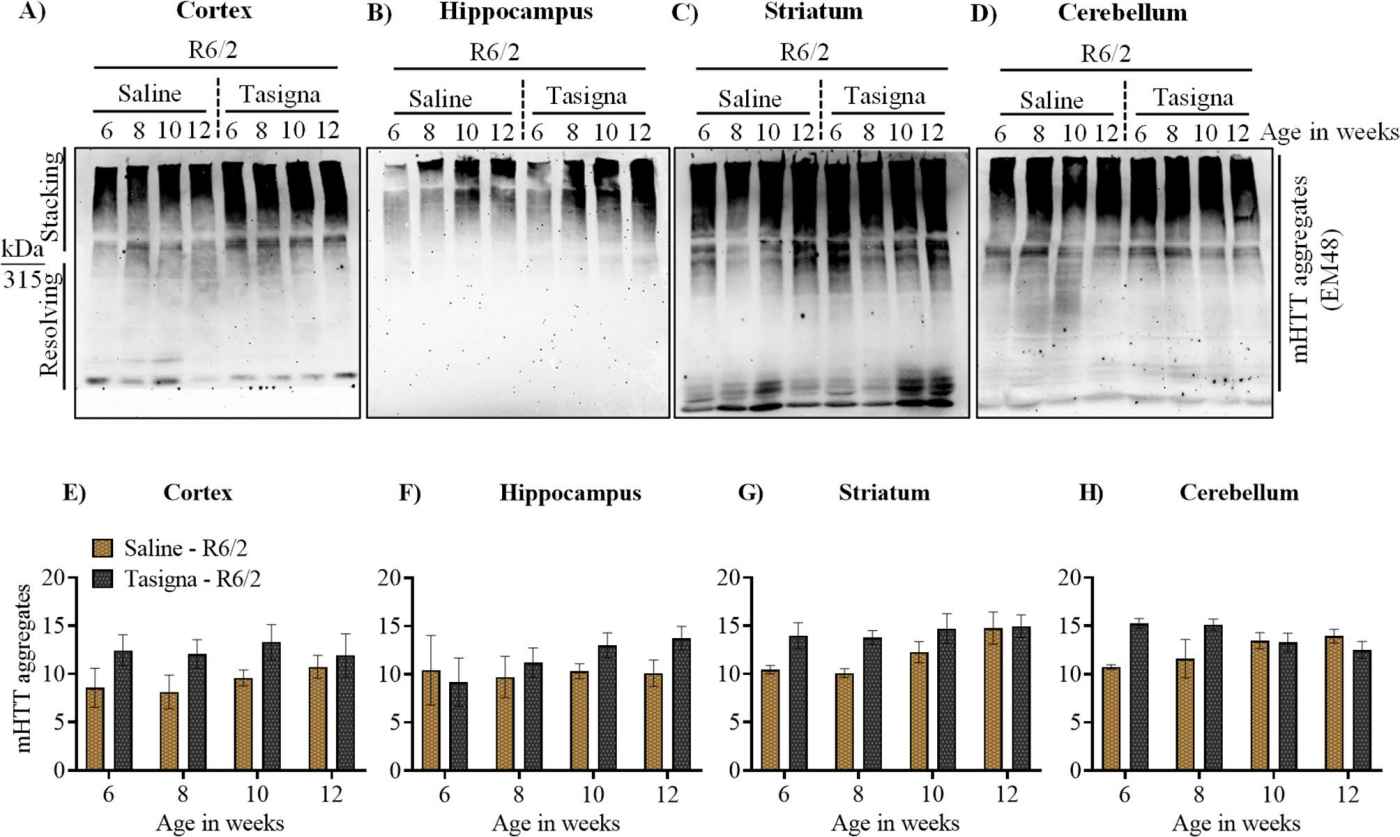
Nilotinib (Tasigna™) is ineffective in enhancing the clearance of mHTT aggregates at any given stage of disease progression in R6/2. (**A**)–(**D**) Representative immunoblot showing no effect of Tasigna in clearing mHTT aggregates (EM48) from 6, 8, 10, and 12 weeks in the cortex, Hippocampus, striatum and cerebellum, respectively. (**E**)–(**H**) Pooled quantified bar graphs for mHTT aggregates for cortex: Age x treatment (F_(3,12)_ = 0.26, p = 0.84), hippocampus: Age x treatment (F_(3,12)_ = 0.49, p = 0.69), striatum: Age x treatment (F_(3,12)_ = 1.27, p = 0.32) , and cerebellum Age x treatment (F_(3,12)_ = 4.603, p = 0.02). N = 3 (R6/2-Saline), and (R6/2-Tasigna) for all the 4 age groups 6, 8, 10, and 12 weeks. Error bars indicate ± SEM. (Mean and ± SEM values are represented in supplementary Table 6). N = number of mice. The treatment groups are separated by vertical dashed lines. Statistical analysis was done by Two-way ANOVA followed by Bonferroni *post-hoc* test. Image brightness and contrast were adjusted for representative purpose. Blots with different exposures and full uncropped raw blots are represented in the supplementary Figs. S4 and S12, respectively.

**Figure 8:**
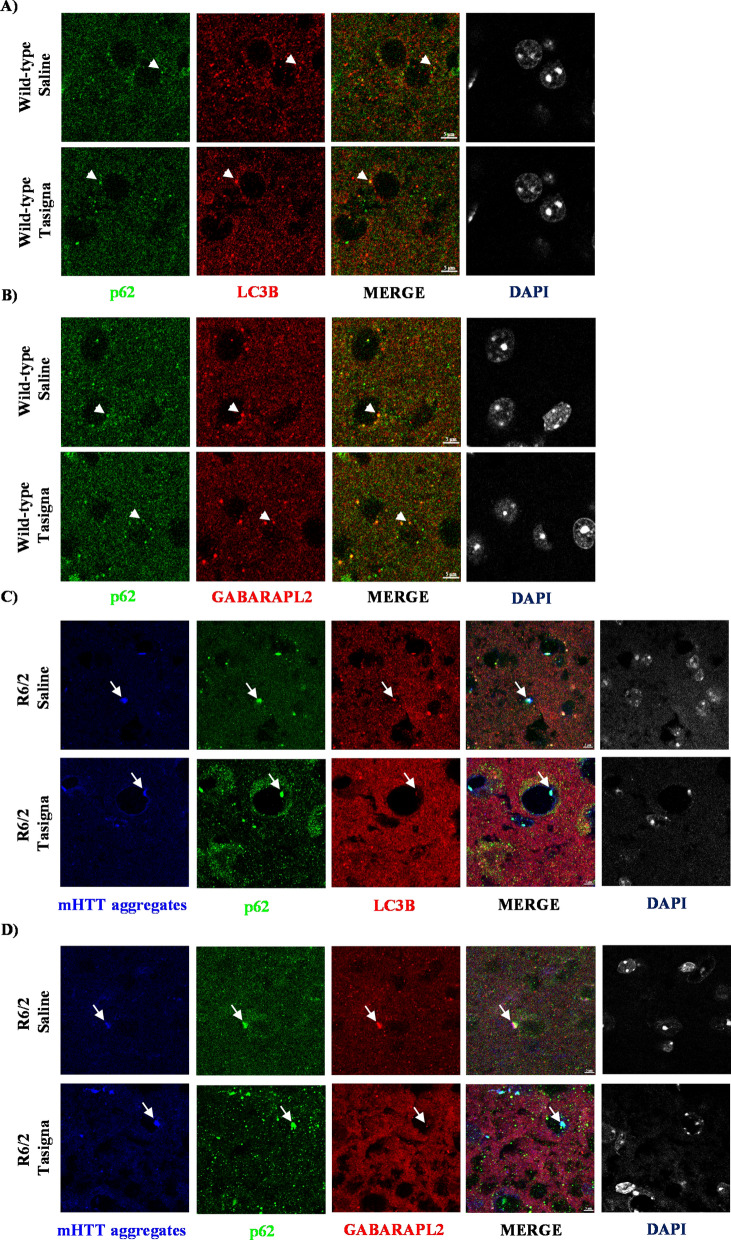
Nilotinib (Tasigna™) does not affect the expression of ATG proteins and clearance of mHTT aggregates at 12 weeks in Striatum in R6/2. (**A**) Representative images of Immunohistochemistry for labelling ATG proteins p62/SQSTM1, LC3B from WT-Saline and WT-Tasigna treated mice in the striatum at 12 weeks. p62/SQSTM1 labelled in green, LC3B in red. (**B**) Representative images of Immunohistochemistry for labelling ATG proteins p62/SQSTM1, GABARAPL2 from WT-Saline and WT-Tasigna treated mice in the striatum at 12 weeks. p62/SQSTM1 labelled in green, GABARAPL2 in red. (**C**) Representative images for Immunohistochemistry for labelling mHTT aggregates by EM48, and ATG proteins p62/SQSTM1 LC3B from R6/2-Saline and R6/2-Tasigna treated mice in the striatum at 12 weeks. mHTT (EM48) aggregates are labelled in Blue, p62/SQSTM1 in green, and LC3B in red. (**D**) Representative images for Immunohistochemistry for labelling of mHTT aggregates by EM48, and ATG proteins p62/SQSTM1, and GABARAPL2 from R6/2-Saline and R6/2-Tasigna treated mice in the striatum at 12 weeks. mHTT (EM48) aggregates are labelled in Blue, p62/SQSTM1 in green, and GABARAPL2 in red. Magnification = 63X. Scale bar = 5 μm. N = 3 WT-Saline, and WT-Tasigna; N = 3 R6/2-Saline, and R6/2-Tasigna. N = number of mice, White arrowheads indicate co-localisation of p62/SQSTM1 with LC3B or GABARAPL2. White arrow indicates co-localisation of mHTT (EM48) aggregates with ATG markers.

Despite the inability of Tasigna in inducing autophagy in R6/2 and wild-type control mice and given the accelerated disease progression in R6/2, it is possible that Tasigna may increase mHTT aggregates clearance to an extent. Results of immunoblot assays further validated our earlier observations on the inability of Tasigna to clear mHTT aggregates in Tasigna treated group compared to saline across all four age groups in R6/2 in the cortex (Fig. 7A,E; Fig. S4 A–D), Hippocampus (Fig. 7B,F; Fig. S4 E–H), striatum (Fig. 7C,G; Fig. S4 I–L), and cerebellum (Fig. 7D,H; Fig. S4 M–P). We did not check for the expression of mHTT in wild-type as the antibody detects only the mutant protein. In addition, we investigated whether administration of Tasigna modified global UBIQUITINATION profile of different brain regions in R6/2 and wild-type control mice. We observed that there was no significant change observed in the UBIQUITINATION pattern in Tasigna treated group compared to saline in wild-type control mice in all the age groups in the cortex (see supplementary Figs. S5A,E), Hippocampus (see supplementary Fig. S5B,F), striatum (see supplementary Fig. S5C,G), and cerebellum (see supplementary Fig. S5D,H), except for 8th week, where a significant decrease was observed in Tasigna treated group in the cerebellum. Similarly, Tasigna failed to change the ubiquitination pattern irrespective of the stage of disease progression in all the age groups in R6/2 in the cortex (see supplementary Fig. S6A,E), Hippocampus (see supplementary Fig. S6B,F), striatum (see supplementary Fig. S6C,G), and cerebellum (see supplementary Fig. S6D,H). For Immunohistochemistry, however, we looked into the expression of autophagy markers in the striata of both R6/2 and wild-type control mice at 12 weeks. Tasigna did not modulate the expression of ATG proteins p62/SQSTM1, LC3B, and GABARAPL2 at 12 weeks in the striatum in wild-type control mice (Fig. 8A,B). Moreover, as Tasigna did not induce autophagy in R6/2, there was no change observed in the expression of p62/SQSTM1, LC3B, and GABARAPL2 in Tasigna treated group compared to saline-treated at 12 weeks in the striatum (Fig. 8C,D). When compared to the saline-treated group, Tasigna treatment did not have any effect on the clearance of mHTT aggregates in R6/2 (Fig. 8C,D). Overall, our results suggest that Tasigna was ineffective in clearing the mHTT aggregates at any given stage of disease progression, thereby it was unable to improve the motor functions in R6/2.

## Discussion

Neurodegenerative diseases are severe forms of neurological aberrations due to progressive degeneration of neurons. Studies have shown that accumulation of misfolded proteins such as β-AMYLOID, α-SYNUCLEIN, and HUNTINGTIN are due to the defects in protein homeostasis, and is the primary cause for many age-related neurodegenerative diseases^1,7^. Therefore, maintaining proteostasis is essential for healthy neuronal connections, which facilitates normal motor functions and coordination. There are many pieces of evidence suggesting that most neurodegenerative diseases arise due to abnormal protein dynamics with defects in protein degradation pathways, leading to accumulation of toxic intracellular and intranuclear aggregates^6,54,55^. It is further supported by the fact that intra-neuronal aggregates of many misfolded proteins are the substrates for autophagic degradation^56,57^. However, HD is a special case with regard to misfolded protein clearance via autophagy. It is well-established that HTT is essential for neuronal survival and plays a role in autophagy as a scaffold protein and in the initiation complex^58–60^. Therefore, understanding the role of autophagy in HD pathogenesis and finding a suitable autophagy modulator is challenging.

In this study, we used the HD mouse model, R6/2 to study the proteostasis defects concerning pathological significance in autophagy dysfunction. We detected mHTT aggregates at a very early stage of disease progression, i.e. from 2 weeks in cortex and striatum, and 4 weeks in Hippocampus and cerebellum suggesting that striatum and cortex are affected primarily leading to disruption in cortico-striatal pathways, thereby initially impairing cognitive functions such as attention, mood and other executive and motor functions later in Huntington’s disease^61,62^. Next, we asked whether UBIQUITIN expression profile is altered as mHTT aggregates are labelled by UBIQUITIN and degraded by macroautophagy^63^. The expression of UBIQUITIN profile in R6/2 was increased in both stacking and resolving in the region striatum at 12 weeks when compared to wild-type, suggesting that there was no defect in the labelling of mHTT aggregates by UBIQUITIN molecules.

Studies have shown that autophagy in the brain is tightly controlled and regulated, and difficult to modulate autophagy flux inside the neuronal cells^46,47^. To understand autophagy regulation in the R6/2 mouse brain, the basal level of autophagy was studied by investigating the expression level of key ATG proteins such as p62/SQSTM1, LC3B and GABARAPL2 from the cortex, Hippocampus, striatum, and cerebellum across different stages of disease progression, in the soluble fraction lysates, and found no significant change in the overall expression level of any of these proteins. Despite the accumulation of toxic mHTT aggregates in the soluble fraction lysates of different regions, the basal level autophagy was not altered in R6/2 in the same fraction, which is in corroboration with another study that showed no alteration in basal autophagy despite the metabolic dysfunction^48^. However, contrary to an earlier study, we comprehensively examined basal autophagy levels in major regions of the brain during various stages of disease progression in R6/2. We assayed the expression of ATG proteins and accumulation of mHTT aggregates only in the soluble fractions because soluble fractions are toxic and disrupt many cellular processes leading to pathogenicity in HD^64–69^. There are other few studies, which propose that insoluble aggregates formed as inclusion bodies are neuroprotective. One of the landmark paper published in Nature from Steve Finkbeiner group has shown that the formation of inclusion bodies reduces the toxicity and load of mHTT aggregates, thereby protecting the neurons from dying^70^. In support of this, few groups have also shown the neuroprotective role of mHTT aggregates in inclusion bodies^71,72^. Some of the studies contradict the function of inclusion bodies as neuroprotective in HD^73–76^ Contemplating these results, the role of mHTT aggregates as inclusion bodies in HD is debatable and inconclusive; therefore further studies have to be carried out to understand its role in neurodegenerative diseases. Our study hypothesises that the steady-state basal autophagy flux in neurons does not cope with the rapid build-up of mHTT aggregates and the rate at which aggregates get cleared by basal autophagy is not sufficient to maintain protein homeostasis to prevent neuronal death. Another plausible hypothesis for autophagic dysfunction without the protein level being affected involves a deficit in the functionalities of ATG proteins, rather than in their levels. This hypothesis supports our findings as there is a significant change in the expression of LC3B-I form in the cortex at 12 weeks, suggesting a possible defect in the conversion rate of LC3B, suggesting aggrephagy dysfunction in R6/2 mice. Another possible hypothesis is impairment in the expression of ATG genes and the studies shown so far has conflicting results. Considering the fact that HTT plays a role in autophagy pathway^77–80^, it is of no surprise that in HD patients, there is clear evidence that gene expression of the autophagy pathway is altered^81^. In one of the studies, the expression levels of autophagy mRNA and proteins are unaltered in HD mouse model despite the presence of metabolic dysfunction^82^. In this regard, a comprehensive analysis of gene expression of various ATG proteins needs to be elucidated across different stages of disease progression in R6/2 mice. Given the fact that mHTT aggregates are dynamic and exist in multiple conformations inside the neurons, studies have shown that many ATG proteins get sequestered by aggregates into its hydrophobic core, thereby causing autophagy dysfunction^42,83–85^. Thus, inducing autophagy at an early stage of disease progression might be beneficial, where newly expressed, functional ATG proteins might potentially enhance the clearance of mHTT aggregates in neurons.

The HD mouse model, R6/2, was one of the first and extensively used models in pre-clinical drug trials to target autophagy^28^. Inducing autophagy was proven to be beneficial in clearing the toxic aggregates, thereby rescuing the behavioural phenotypes and increasing the lifespan in several neurodegenerative diseases^8,86–89^. Small molecule modulators of autophagy are effective in inducing the process in several models of neurodegenerative diseases, including HD^86,90^. Our lab has previously shown that inducing autophagy was proven to be beneficial in clearing toxic α-SYNUCLEIN aggregates, thereby rescuing behavioural phenotypes in a pre-clinical mouse model of PD^8,91^. Nilotinib (Tasigna™) is a BCR-ABL receptor tyrosine kinase inhibitor and was proved to be effective in the clearance of α-SYNUCLEIN aggregates by inducing autophagy, thereby preventing neurons from dying, and improving the motor functions in MPTP mouse model of PD^30,31^. Currently, Tasigna is in clinical trials to study the tolerance of the drug in PD, and HD patients, although not validated using a pre-clinical model. Therefore, we tested the efficacy of Tasigna in the more severe and robust form of the HD mouse model, R6/2. These mice develop normally until 6–8 weeks of age but exhibit neurological symptoms similar to HD patients such as hind-limb clasping, tremor, grooming, poor coat condition, dyskinesia, irregular gait, and motor dysfunction, with eventual death by 12–13 weeks, making it an extremely robust system of HD^29,15,92^. Our results from a battery of behavioural tests suggest that Tasigna is ineffective in rescuing neither the motor functions and body weight nor extend lifespan in R6/2 mice.

We speculate based on our behavioural results that there might be several reasons accounting for discrepancies observed regarding a model-based bias ineffectiveness. Perhaps, due to the aggressive and penetrant phenotypes of R6/2, subtle improvements caused by Tasigna treatment at the molecular level may mask any observable changes in behaviour. However, surprisingly, given that Tasigna is in Phase-I clinical trials for HD, we found no effect of Tasigna in inducing autophagy. Thus, the expression of key ATG proteins remained unaltered in R6/2. Moreover, due to this failure to induce autophagy, there was no difference in the clearance of mHTT aggregates in the soluble fractions at any stage of disease progression in R6/2. However, our study investigated only the soluble fractions of lysate in the view of soluble aggregates are toxic and causes neuronal death. The levels of the aggregates and the ATG proteins present in the insoluble fractions remains to be elucidated. Further to shed light on the effect of Tasigna on the recruitment of ATG proteins onto the mHTT aggregates, image analyses at the early stages of disease progression needs to be done. Given the lack of detailed investigations related to the effect of Tasigna in inducing autophagy and based on a study shown that Tasigna induces autophagy unconventionally through a non-canonical pathway in hepatocellular carcinoma cells^93^, a detailed investigation is required to ascertain the use of Tasigna in HD patients by using appropriate pre-clinical models. A thorough examination of the expression of autophagy genes also needs to be carried out to conclusively assert the effects of Tasigna in pre-clinical models of HD. It is possible that dosage optimization might give an insight into the efficacy of this drug in inducing autophagy, but however, it is also plausible that Tasigna effectively induces autophagy in the presymptomatic stage when the aggregate load is not overwhelming. For therapeutic benefit, the desired levels of Tasigna in inducing autophagy in R6/2 mice may not be achieved by the concentration used in this study, due to the severity and rapid progression of the disease. Studying the effect of Tasigna at higher concentrations could throw clarity on the appropriate dosage and effectiveness of the drug. Studies so far carried out to exhibit the effect of Tasigna as an active therapeutic molecule for the neurodegenerative disease is in concern with C-ABL inhibition and some studies are also focused on autophagic clearance of α-SYNUCLEIN and amyloid aggregates in Parkinson’s and Alzheimer’s disease respectively. It has been previously established that C-ABL kinase levels are upregulated in Parkinson’s disease^94–96^. However, concerning Huntington’s disease, no reports of over-expression of C-ABL and its interaction with HTT protein in any of the HD mouse models to date. The levels of C-ABL kinase in HD and its modulation after treatment with Tasigna needs to be elucidated. Apart from R6/2, many HD mouse models have been developed by expressing full-length *HTT* gene containing a variable number of polyQ repeats such as BACHD, YAC128 transgenic mouse models or CAG140, CAG150 knock-in mouse models^97–99^. These mouse models exhibit less severe and modest behaviour phenotypes^100^. Moreover, since R6/2 expresses only exon 1, but not complete *HTT* gene, it is often considered as polyglutamine toxicity model rather than HD model. However, pathogencity and symptoms in R6/2 are correlated with juvenile HD. So, considering R6/2 as a model system for HD or for polyglutamine toxicity is a debatable topic. Therefore, We hypothesize that Tasigna, when tested in these mouse models or at a higher dose, may prove to be effective in rescuing the motor functions and ameliorate the disease phenotype.

## Methods

### Ethics statement

This study was carried out according to the guidelines of Institutional Animal Ethical Committee (IAEC), and Committee for Control and Supervision of Experiments on Animals (CPCSEA). All the protocols and experiments used in this study were approved by institutional animal care committee at Jawaharlal Nehru Centre for Advanced Scientific Research (JNCASR, Bangalore), and all the experiments were carried out in accordance with relevant guidelines and regulations.

### Mouse studies

All experimental mice were maintained and bred in the animal house facility at JNCASR under 12-h light and dark cycle. Food and water were available ad libitum*.* All the experiments were performed according to the guidelines of Institutional Animal Ethical Committee (IAEC), and Committee for Control and Supervision of Experiments on Animals (CPCSEA). Huntington’s disease mouse model, R6/2 (B6CBA-Tg (HDexon1) 62Gpb/3 J; https://www.jax.org/strain/006494), procured from Prof. Nihar Ranjan Jana, NBRC, New Delhi, (approved by Jackson’s laboratory) and bred with C57BL/6 wild-type mice in 1:3 ratio respectively. Hemizygous R6/2 females were infertile, and hence, not used for breeding. Animals were genotyped for R6/2 and wild-type control mice. Following primers were used to determine the genotype for R6/2: Forward primer: 5′ CCG CTC AGG TTC TGC TTT TA 3′; Reverse primer: 5′ TGG AAG GAC TTG AGG GAC TC 3′. Experimenters were not blinded to the genotype and injection regimen used in the study.

### Injection regimen

Nilotinib (Tasigna™), procured commercially from pharmacy stores, dissolved in saline and mice were injected intraperitoneally every day, from 2 to 12 weeks of age at a dosage of 20 mg/kg body weight in R6/2, and wild-type control mice. Control littermates were injected with saline for both the genotypes.

### Bodyweight measurement and survival

All the experimental mice were weighed using a weighing machine (Cat# DS-450 series, Essae-Teraoka, India) on alternate days before the start of injections, and dosage was calculated according to the weight of the mouse. Bodyweight across weeks was plotted by averaging weights measured on three alternate days.

### Behavioural studies

All the behavioural experiments were done in the behaviour room in the Institute’s animal facility. HD mouse model, R6/2, and wild-type littermates (all males) were used for the experiments from 5–6 to 12–13 weeks of age. Mice used for the behaviour were habituated in the behaviour room for approximately 30 min before the start of the experiment (tests were done once per week). The light intensity was maintained at 100 lx.

#### Open field test

Open Field Test was done to study the locomotion and exploratory behaviour of animals. Open field arena of 50 cm × 50 cm × 45 cm was custom-made at JNCASR using plywood, and the internal surface was coated with white polish. During testing, the mouse was allowed to explore the arena for 5 min by introducing the mouse in the periphery zone, and movement was recorded using a SONY video camera (Model no. SSC-G118, India). The mouse was then returned to its home cage. The open-field arena was cleaned with 70% ethanol and air-dried before testing the next mouse. Distance travelled was calculated off-line by the experimenter using SMART v3.0.04 software (Panlab Harvard Apparatus, U.S.A).

#### Rotarod test

The Rotarod instrument was custom-made at the mechanical workshop at National Centre for Biological Sciences, Bengaluru, India. The diameter of the rotating rod was 3.3 cm made of Delrin and textured to enhance the grip of the animal. The rotating rod was fixed at the height of 30 cm from the platform, and the rod was partitioned into 3 areas at 9.3 cm gap between each partition using discs made of Teflon of 40 cm diameter. Mice were trained for 5 consecutive days at different revolutions per minute (rpm) accelerated at 1 rpm/5 s, except on day-4 and -5, for 3 trials. Mice were trained at 5–10 rpm on day-1, 11–15 rpm on day-2, 16–20 rpm on day-3, and 20 rpm on day-4 and -5. During testing, mice were given 3 trials at 15 rpm for 60 s. After the trial/testing, each mouse was returned to their respective home cage. The rotating rod was wiped with 70% ethanol and was allowed to dry before testing the next animal. All the trials were recorded using a Sony HD camera (HDR-CX405), and videos were analysed to score the latency to fall manually. Latency to fall was calculated by averaging the time spent on the rotarod from three trials.

#### Hind-limb clasping

Hind-limb clasping was performed to assess the motor coordination and stages of disease progression. Mice were suspended in the air by holding its tail for a maximum of 60 s and recorded using a Sony HD camera (HDR-CX405) and analysed manually. Depending on its hind-limb movements, scores were calculated as follows: Score zero = Hind-limb splayed outward, away from the abdomen; Score one = One hind-limb retracted towards the abdomen (50% of the time); Score two = both hind-limbs retracted towards the abdomen (50% of the time); Score three = both hind-limbs entirely retracted and touching the abdomen. Animals were further scored based on the time taken to clasp: Score Zero = No clasping; Score one = Clasping in 31–60 s; Score two = Clasping in 16–30 s; Score three = Clasping in 11–15 s, Score four = Clasping in 6–10 s; Score five = Clasping in 1–5 s. Lower the score, better the performance of the animal.

### Western blot analysis

Lysates from different regions of the brain such as Cortex, Hippocampus, Striatum, and Cerebellum from wild-type and R6/2 were prepared in modified RIPA (Radio-Immunoprecipitation Assay) lysis buffer: 150 mM NaCl (Cat# S6191, Sigma, India), 50 mM Tris–HCl, pH 7.4 (Tris—Cat# 15965, Fisher Scientific, India; HCl—Cat# HC301585, Merck, India), 5 mM EDTA (Cat# RM1195, HiMedia, India), 0.25% sodium deoxycholate (Cat# D6750-100G, Sigma, India), 0.1% Triton X (Cat# 845, HiMedia, India), 0.1% SDS (Cat# 161-0302, Bio-Rad, India), with protease inhibitor (Cat# 11836170001, Sigma, India), and phosphatase inhibitor cocktails (Cat# P2850—Cocktail 1, P5726—Cocktail 2, P0044—cocktail 3 Sigma, India). Homogenization was done in 7 mL (Cat# D9063, Sigma, India), and 15 mL (Cat# D9938, Sigma, India) Dounce tissue homogenizers. Lysates were centrifuged at 14,000 rpm at 4 °C for 30 min, and the supernatant was collected. Proteins were estimated by Bradford assay using Bradford reagent (Cat# 5000006, Bio-Rad, India), and electrophoresed in SDS-PAGE: 5% stacking used for all the experiments while 12% resolving gels were used for LC3B, p62/SQSTM1, and GABARAPL2, whereas 6% and 8% gels were used for mHTT aggregates (EM48) and UBIQUITIN respectively for ~ 2 h. The separated proteins were transferred onto PVDF (Polyvinylidene fluoride) membrane (Cat# 162-0177, Bio-Rad, India) for 2 or 24 h approximately for ATG-related proteins, and mHTT aggregates at 4 °C respectively. After transfer, blots were blocked in 5% skimmed milk (Cat# M530, Hi-Media, India) for approximately one hour and washed thrice in PBST (Phosphate Buffered Saline Tween; 0.1% Tween-20 (Cat# 28599, Sisco research laboratories, India) in 1X PBS). Proteins were then probed for anti-LC3B raised in rabbit (Cat# L7543, Sigma, India), anti-p62/SQSTM-1 raised in rabbit (Cat# P0067, Sigma), anti-GABARAPL2 raised in rabbit (Cat# PAJ288Hu01, Cloud-Clone, India ), anti-HTT—EM48—raised in mouse (Cat# MAB5374, Merck, India), anti-UBIQUITIN raised in mouse (Cat# P4D1-BML-PW0930-0100, ENZO Life Sciences, India), and anti-β-ACTIN raised in rabbit (Cat# PA116889, Thermo Fischer Scientific). After incubation, blots were washed thrice approximately for 10 min in PBST and incubated in secondary antibodies conjugated with Horse-radish-peroxidase—anti-rabbit (Cat# 1721019, Bio-Rad, India), and anti-mouse (Cat# 1706516, Bio-Rad, India) in 1% skimmed milk approximately for 1 h at room temperature. Proteins were detected using ECL (Enhanced-Luminol-based chemiluminescent substrate; Cat# 1705060, Bio-Rad, India) and visualised using an I-Bright CL1000 Chemidoc system (Thermofisher). Blots were quantified using ImageJ software (v1.52a, National Institutes of Health (NIH), U.S.A).

### Histology

All experimental mice were transcardially perfused with ice-cold Phosphate Buffered Saline (PBS) containing NaH_2_PO_4_.2H_2_O (Cat# 13472-35-0, Fisher Scientific, India), Na_2_HPO_4_.2H_2_O (Cat# 10028-24-7, Fisher Scientific, India), NaCl (Cat# 7647-14-5, Fisher Scientific, India), pH—7.4, and were fixed with 4% paraformaldehyde (PFA) (Cat# GRM3660, HiMedia, India). The flow rate was maintained continuously at 10.2 mL/min using a peristaltic pump (Cat# RH-P110S-25, Ravel Hiteks PVT LTD, India). After perfusion, the tissues were subjected to post-fixation in 4% PFA for a day and preserved in 30% sucrose (with 0.02% sodium azide) (Cat# GRM134, Hi-Media, India) at 4 °C till further usage. Sucrose-treated brains were sectioned in a cryostat (Cat# CM3050s, Leica, India) with Internal chamber temperature (CT) maintained at -20 °C, and Object Temperature (OT) at − 22 °C throughout the procedure. 40 µm thick coronal sections were obtained on gelatin-coated slides (3% gelatin, Cat# GRM019, Hi-Media, India, and 0.5% Chromium potassium sulphate dodecahydrate, Cat# GRM3042, Hi-Media, India). These slides were stored at 4 °C until further usage.

### Immunohistochemistry

For Immunohistochemistry, the unmasking of antigen was done in 10 mM Sodium citrate buffer (Cat# 6132-4-3, Fisher Scientific, India). The sections were then permeabilised with PBSTx (0.1 M PBS and 0.1% Triton-X-100) followed by blocking for ~ 4 h at room temperature with 2% BSA (Bovine Serum Albumin, Cat# 85171, SRL, India) and 1% goat serum, and 1% horse serum diluted in PBSTx. Sections were incubated with primary antibodies for 48 h at 4 °C after 2 h of incubation at room temperature. A cocktail of antibodies was diluted 300 times in 2% BSA prepared in PBSTx. The antibodies used were: anti-LC3B raised in rabbit (Cat# L7543, Sigma, India), or anti-GABARAPL2 raised in rabbit (Cat# PAJ288Hu01, Cloud-Clone, India), anti-p62/SQSTM-1 raised in guinea-pig (Cat# GP62-C, iProgen Scientific), anti-HTT—EM48—raised in mouse (Cat# MAB5374, Merck, India). After primary incubation, sections were probed with secondary antibodies for 4 h at room temperature in the dark. A cocktail of secondary antibodies was diluted 300 times in 2% BSA, 1% goat serum prepared in PBSTx. The secondary antibodies were: Atto Flour 550 goat anti-rabbit (Cat# 43328, Sigma, India), Alexa flour 647 goat anti-guinea pig (Cat# A-21450, Thermo Fisher Scientific, India), and Atto 488 goat anti-mouse (Cat# 62197, Sigma, India. After incubation, sections were mounted with Vecta-Sheild containing DAPI (4′, 6-diamidino-2-phenylindole; Cat# H-1200, Vector Laboratories, CA, U.S.A) and were used for imaging. Images were obtained using confocal microscopy LSM 810 Airy Scan (Zeiss, India), and Delta Vision (29,065,728, API, GE Healthcare Ltd, India).

### Cell culture experiments

HeLa cells were maintained in growth medium composed of DMEM (Dulbecco’s Modified Eagle Medium) (Cat# D5648, Sigma-Aldrich) supplemented with 3.7 g/l sodium bicarbonate (Cat# S5761, Sigma-Aldrich) plus 10% fetal bovine serum (Cat#10270-106, Life Technologies) and 100 U/ml penicillin and streptomycin (Cat#15140-122, Life Technologies) at 5% CO_2_ and 37 °C. Upon confluence, the cells were passaged with 0.05% Trypsin–EDTA (Cat#59418C, Sigma-Aldrich).

The chemicals used in this study for the cell-culture experiments were—Nilotinib (Tasigna™) procured commercially, 6-bromoindirubin-3′-oxime, (6BIO) (Cat# B1686, Sigma-Aldrich, India) and Bafilomycin A1, (BafA1) (Cat#11038, Cayman chemicals).

### Autophagy assay

To perform Autophagy assays, 1.0 × 10^6^ HeLa cells were seeded per well of 6-well plates and were allowed to attach for 24 h. Autophagy was induced using Tasigna™ dissolved in saline (5 µM, 25 µM, 50 µM, 100 µM, 250 µM, and 1 mM), 6 BIO (10 µM) and BafA1 (100 nM) for 2 h.

Following treatments, cells were washed with ice-cold PBS. Cells were then lysed in 100 μl of sample buffer (10% w/v SDS, 10 mM dithiothreitol, 20% vol/vol glycerol, 0.2 M Tris–HCl (pH 6.8), 0.05% w/v bromophenol blue) and then collected using a cell scraper. Western blot was performed to check the expression of LC3B, and a similar procedure was followed, as mentioned in the previous western blot analysis section.

### Statistics

All the graphs were plotted, and statistics were performed using Microsoft Excel 2016, and Graph Pad Prism 8.4 (except for Fig. 4B). Data are presented as Mean ± Standard Error of Mean (SEM). Two-way ANOVA and Three-way ANOVA were applied, followed by appropriate *post-hoc* analysis, to test statistical significance unless otherwise stated.

## Supplementary information

Supplementary information.

## Data Availability

All data related to this study can be obtained on request, while all the analysed data were included in this published article and its supplementary information files.
