# Supplementary material for: Spatiotemporal analysis of soluble aggregates and autophagy markers in the R6/2 mouse model
Source: Sci Rep. 2021 Jan 8;11:96. doi: 10.1038/s41598-020-78850-w (PMC7794371; doi:10.1038/s41598-020-78850-w)
Supplement: Supplementary file 1 — Supplementary information. [file 41598_2020_78850_MOESM1_ESM.pdf]

Spatiotemporal analysis of soluble aggregates and autophagy markers in the R6/2 mouse model

M J Vijay Kumar[1], Devanshi Shah[1#], Mridhula Giridharan[2#], Niraj Yadav[2], Ravi Manjithaya[1,2*], James P Clement[1*]

1. Neuroscience Unit, Jawaharlal Nehru Centre for Advanced Scientific Research, Jakkur, Bangalore, India; 2. Molecular Biology and Genetics Unit, Jawaharlal Nehru Centre for Advanced Scientific Research, Jakkur, Bangalore, India.

E-mail address: 1. vijaykumarmj@jncasr.ac.in; 1#. devanshishah28794@gmail.com; 2#. mridhu.giri@gmail.com; 2. infonrjz5@gmail.com 1, 2*. ravim@jncasr.ac.in; 1*. clement@jncasr.ac.in

#Equal contribution, *Joint corresponding authors.

Supplementary Figure S1
For figure 1

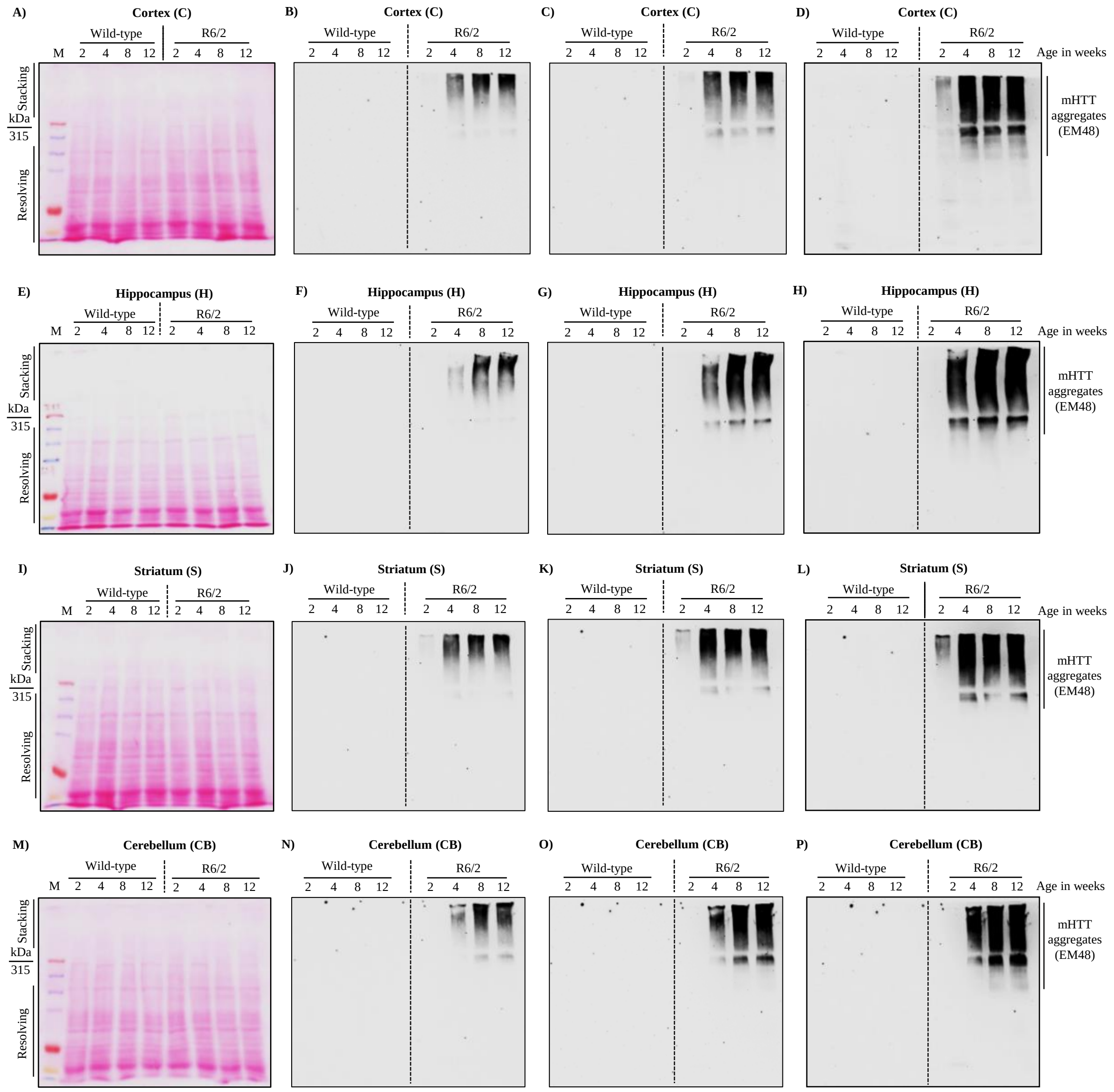

Supplementary figure S2

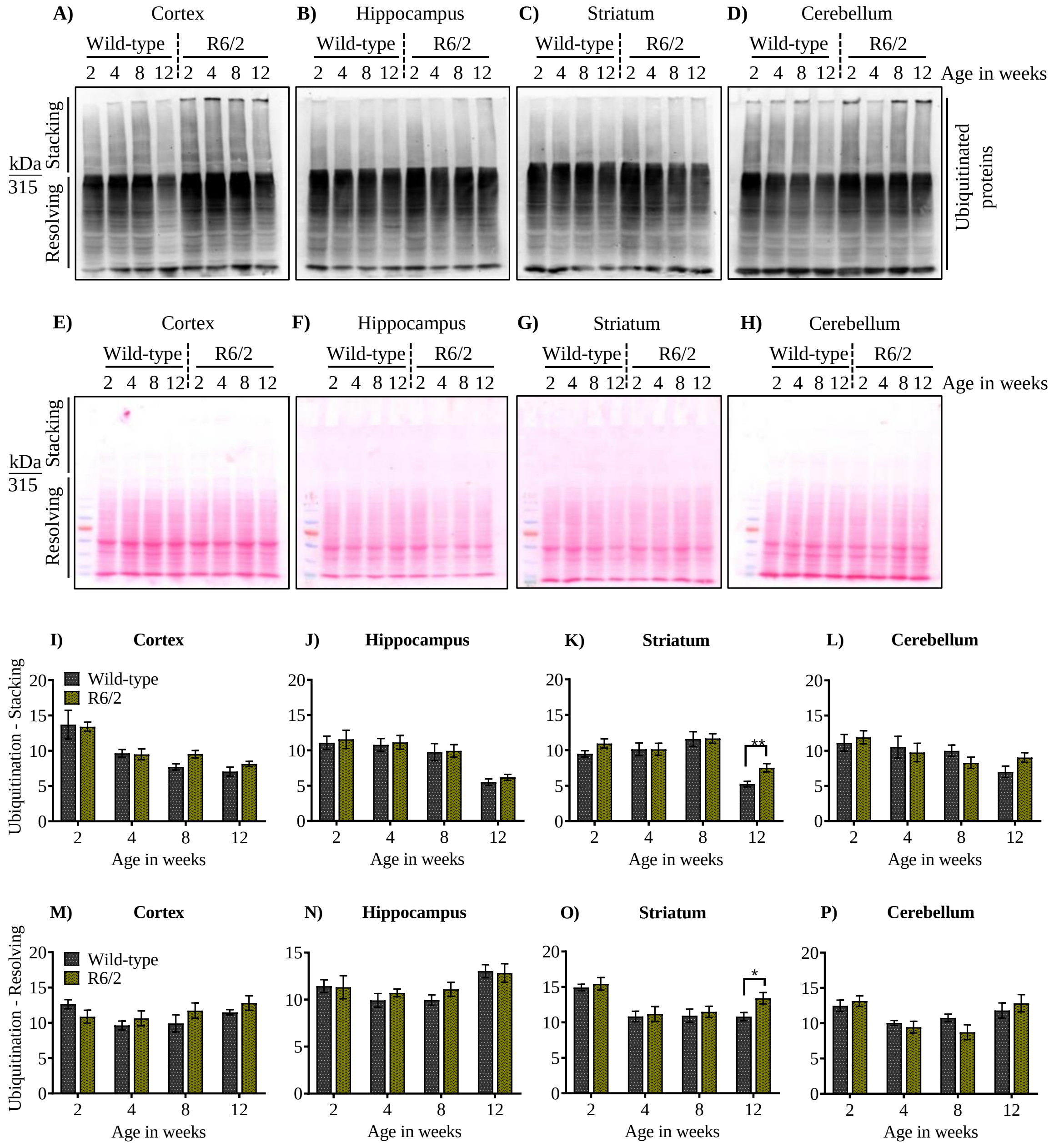

Supplementary figure S3

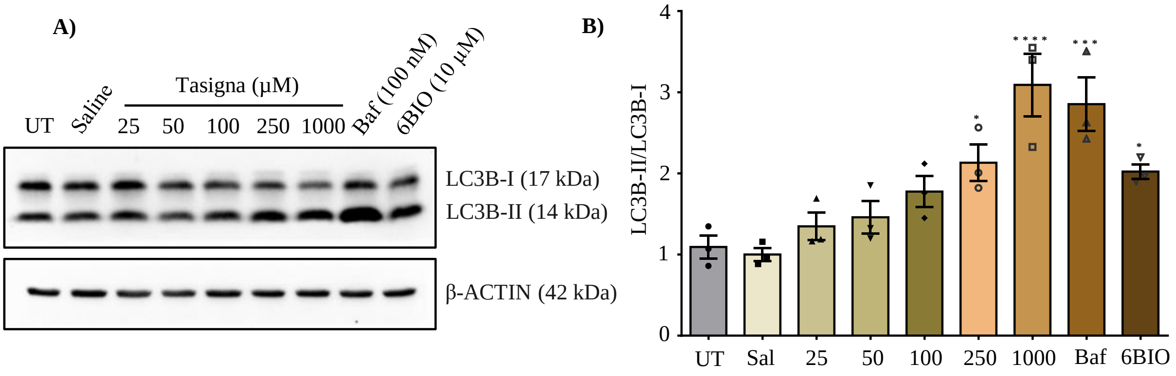

Supplementary Figure S4
For figure 7

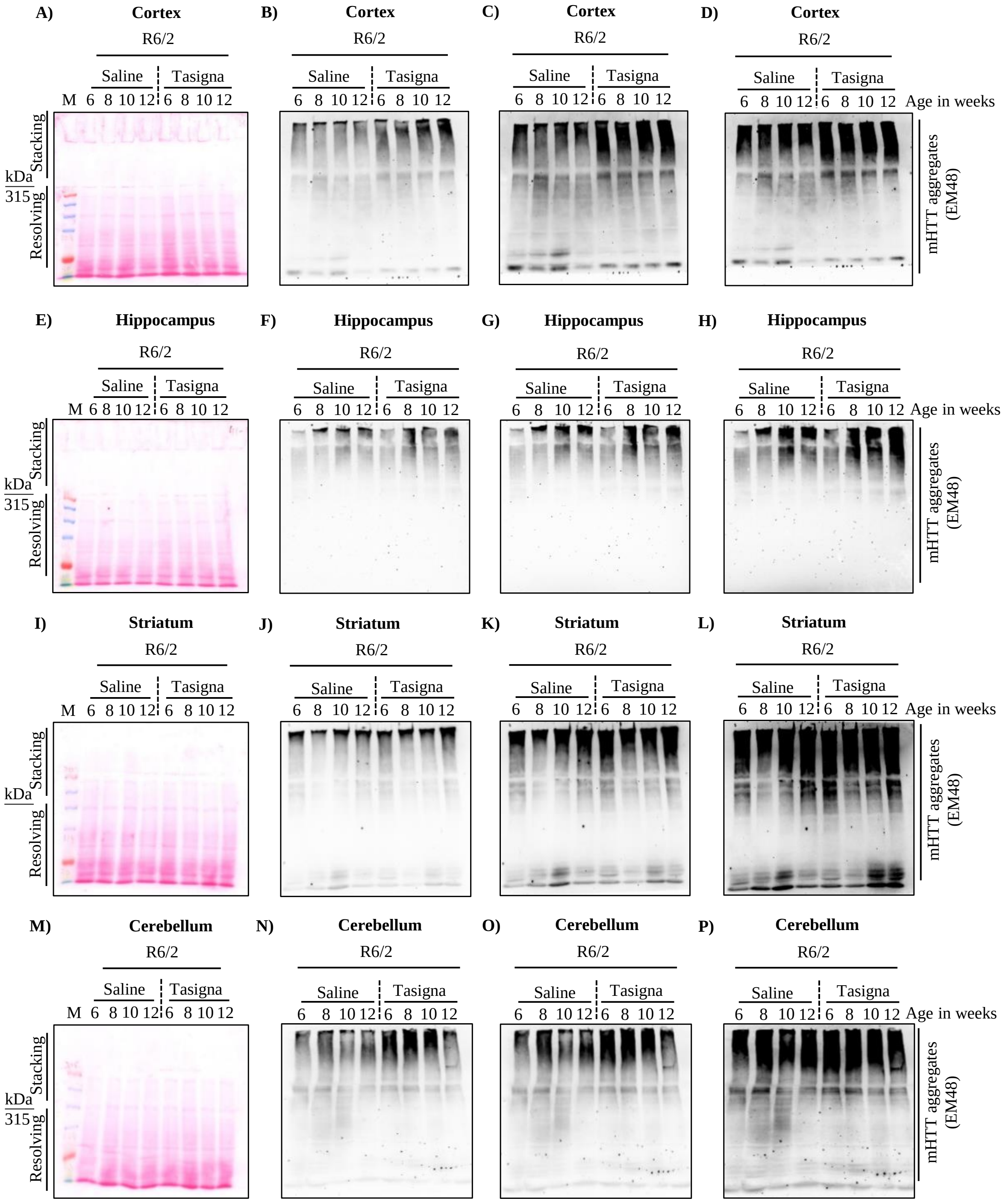

Supplementary figure S5

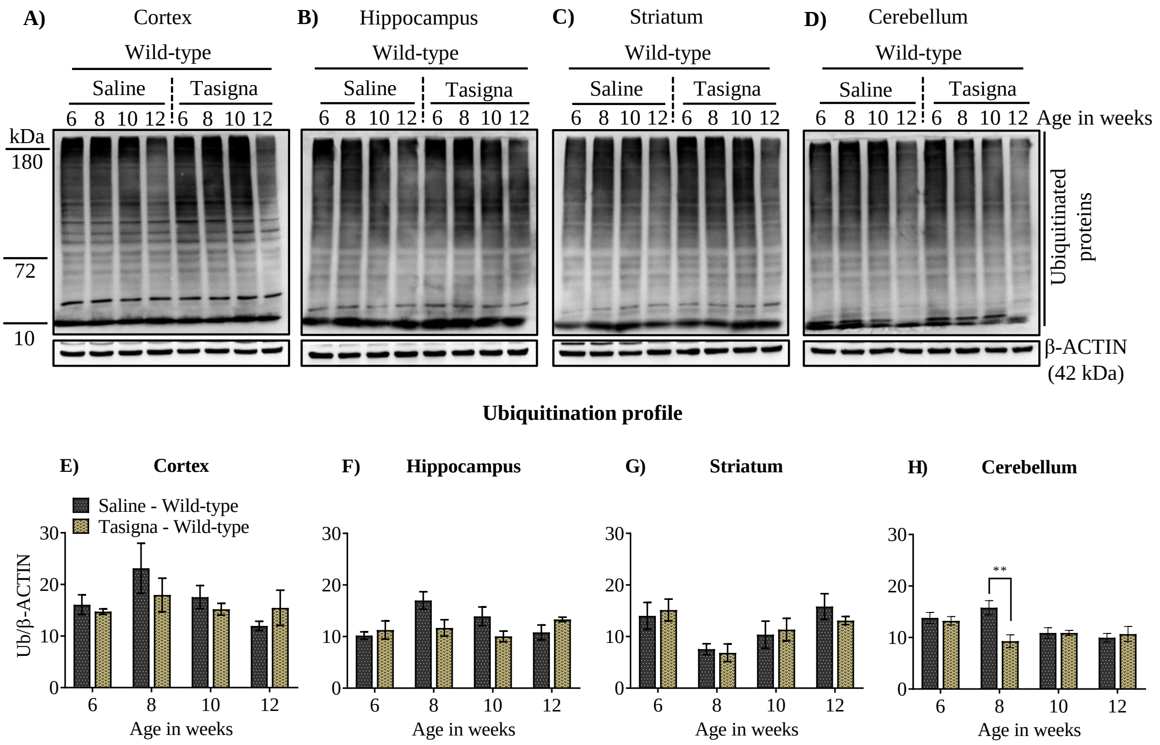

Supplementary figure S6

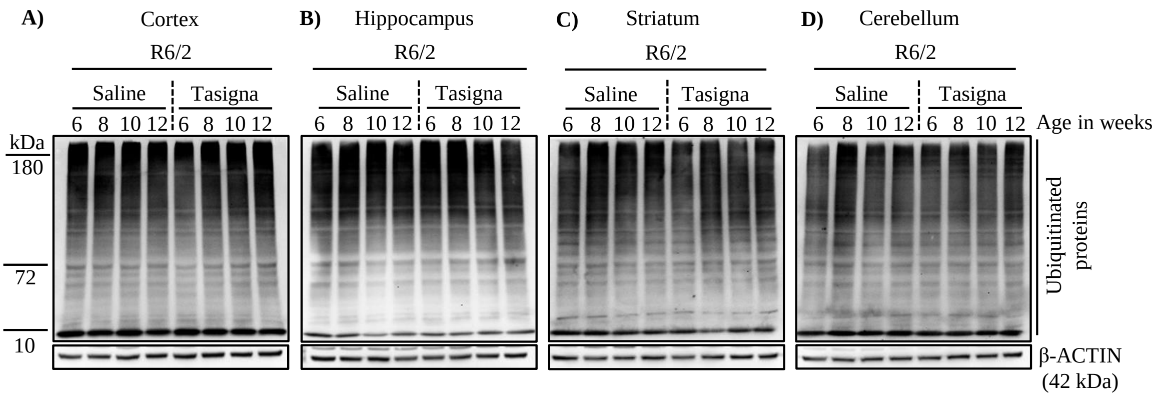

Ubiquitination profile

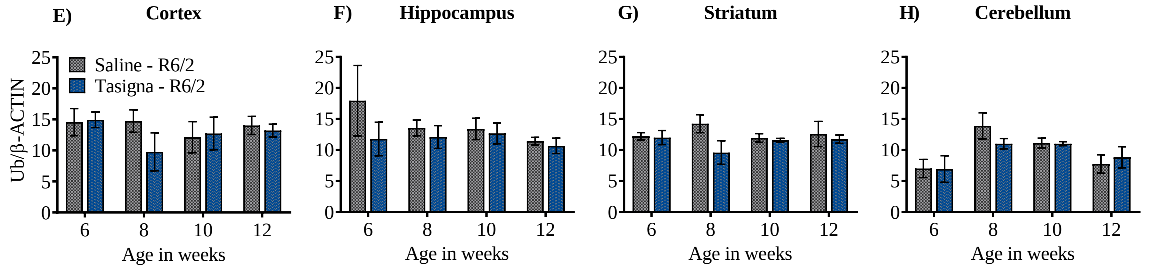

Supplementary figure S7
For Figure 1

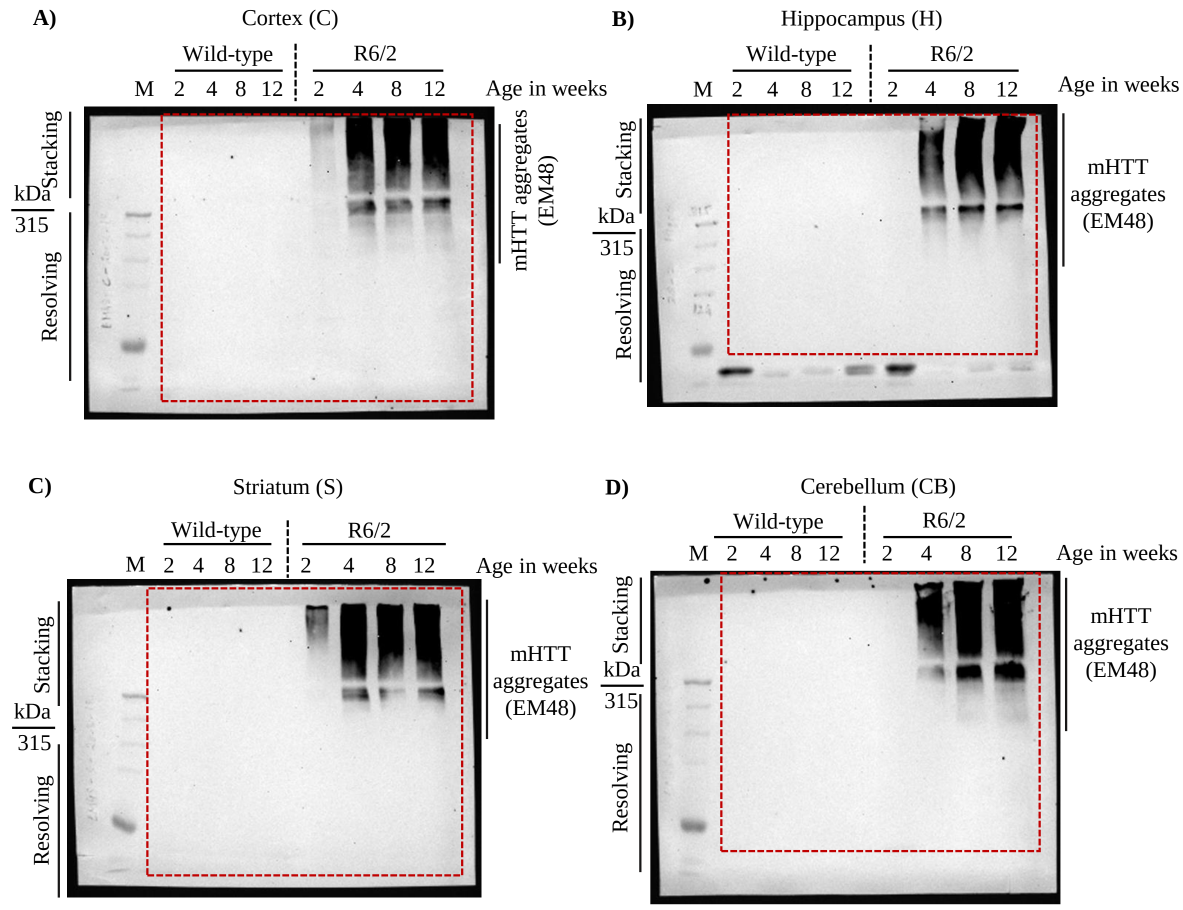

Supplementary figure S8
For Figure 2

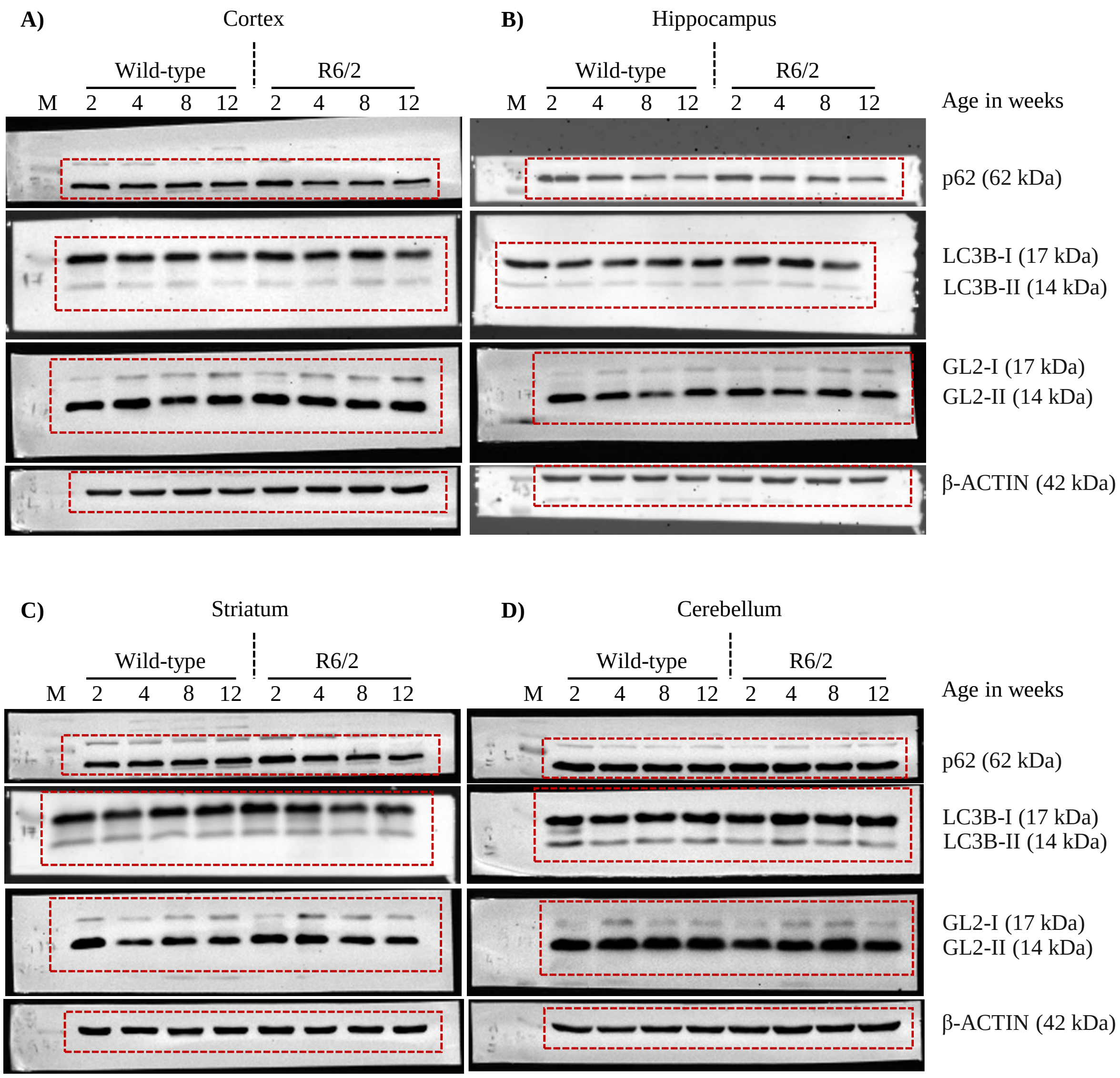

Supplementary figure S9
For figure S2

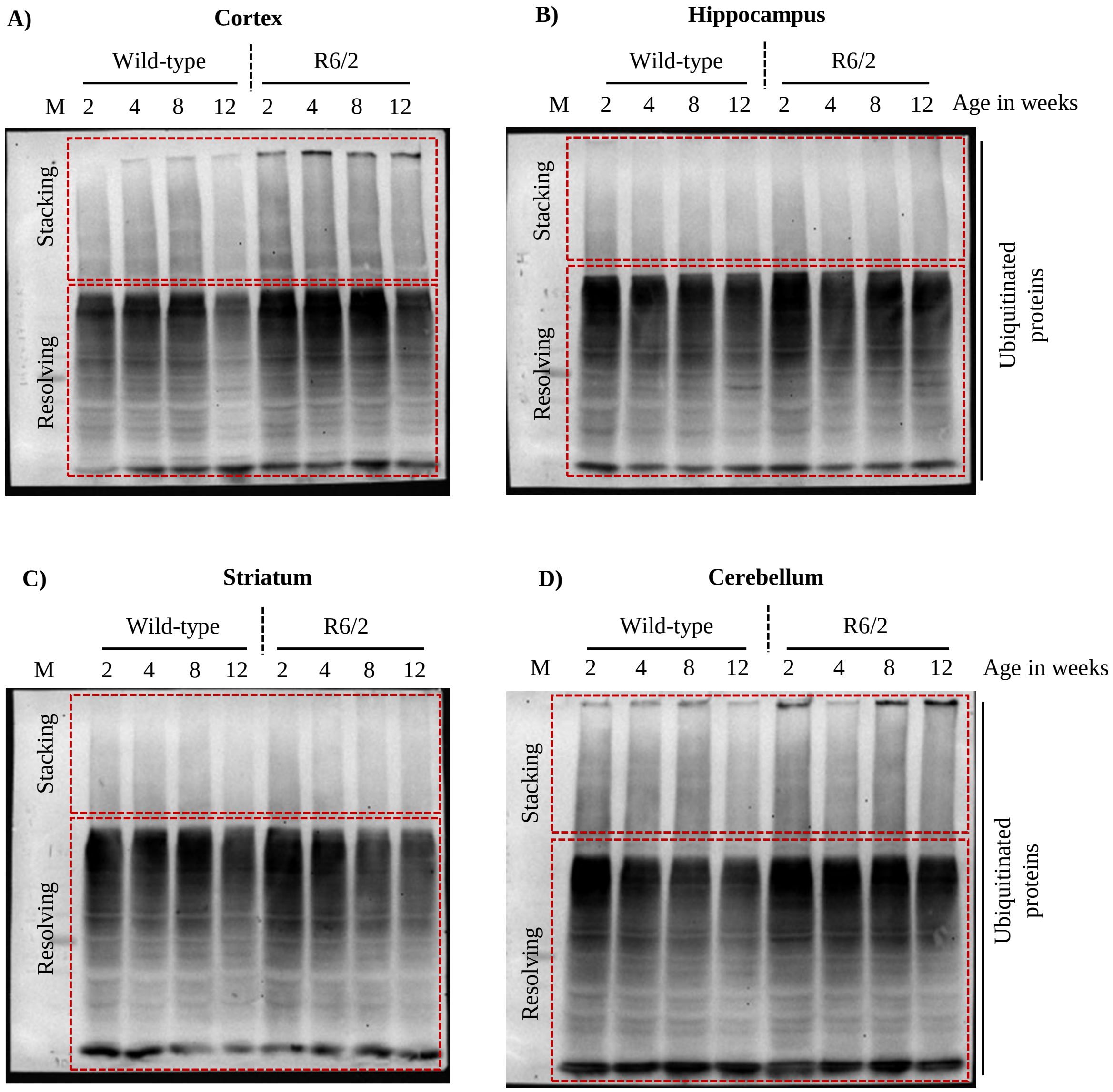

Supplementary figure S10
For Figure 5

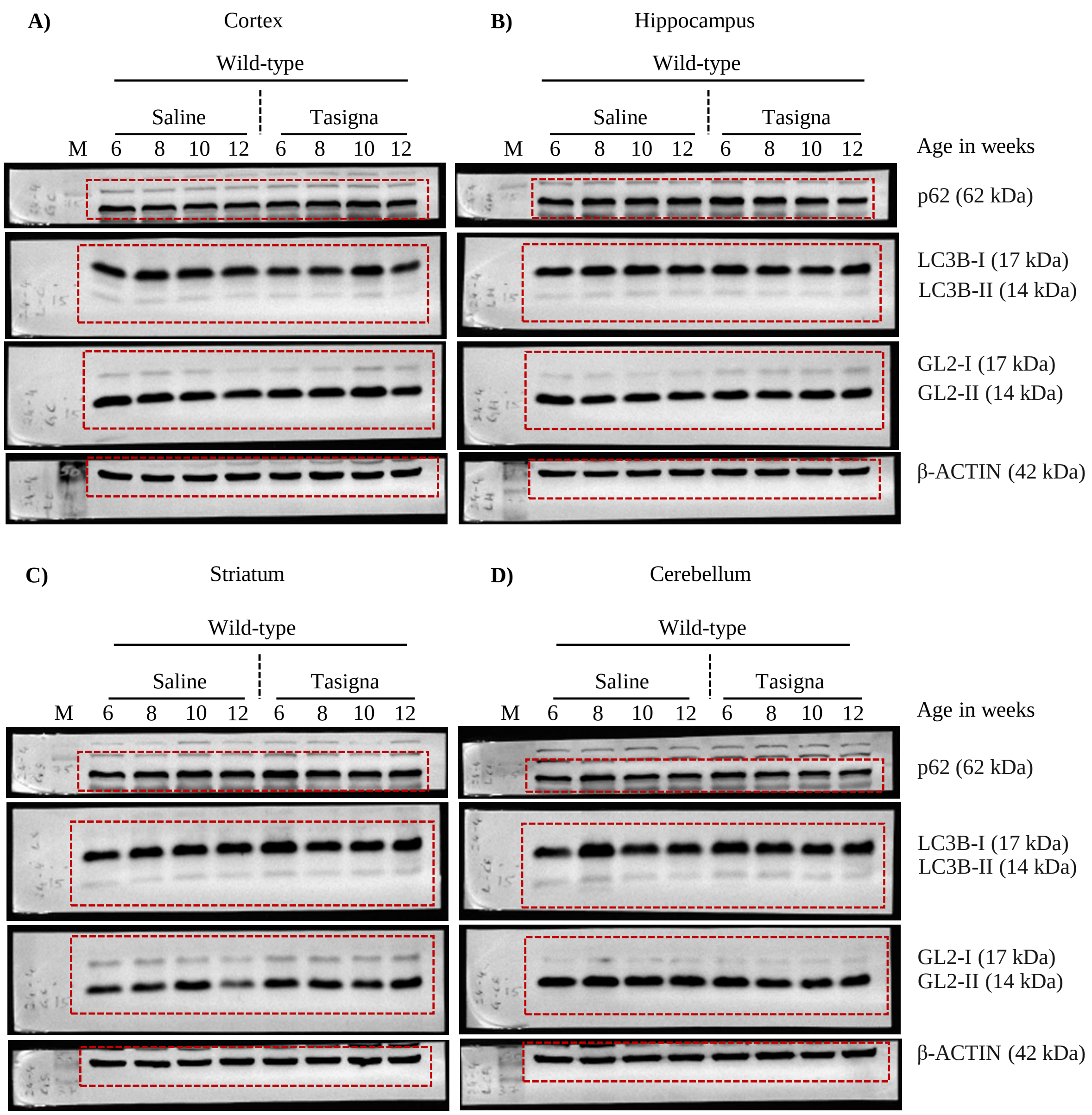

Supplementary figure S11
For figure 6

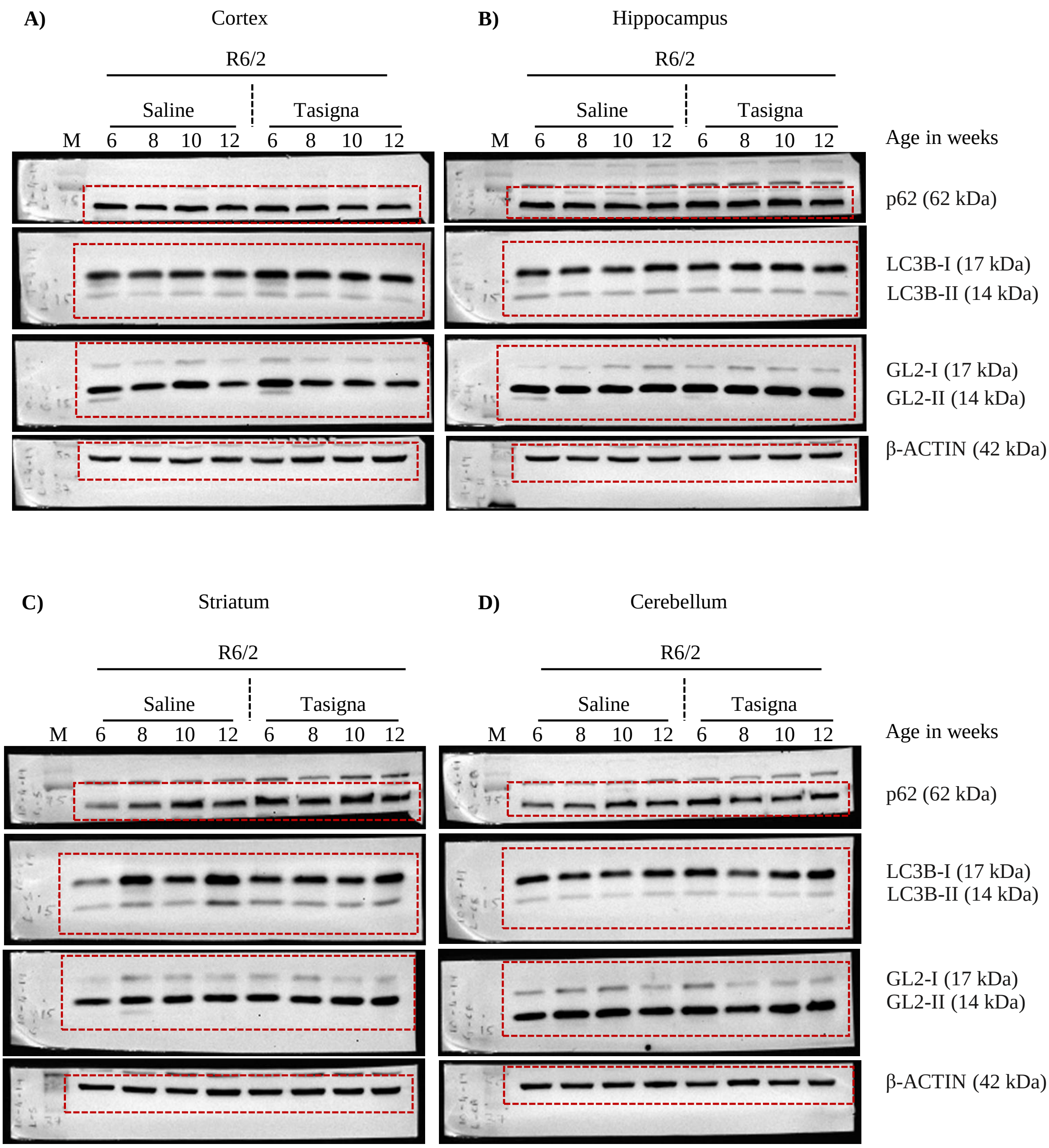

Supplementary figure S12
For figure 7

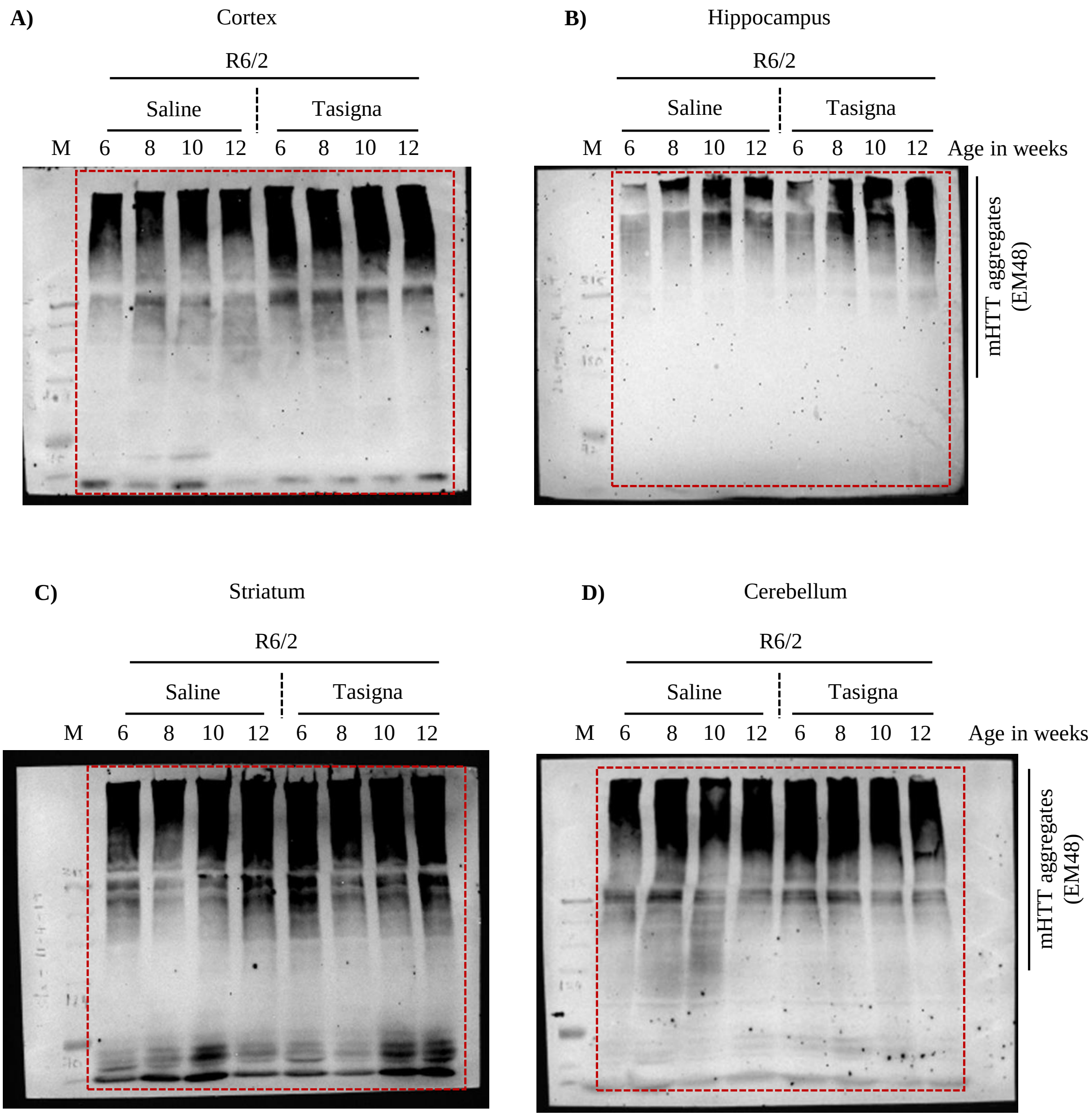

Supplementary figure S13
For Figure S5

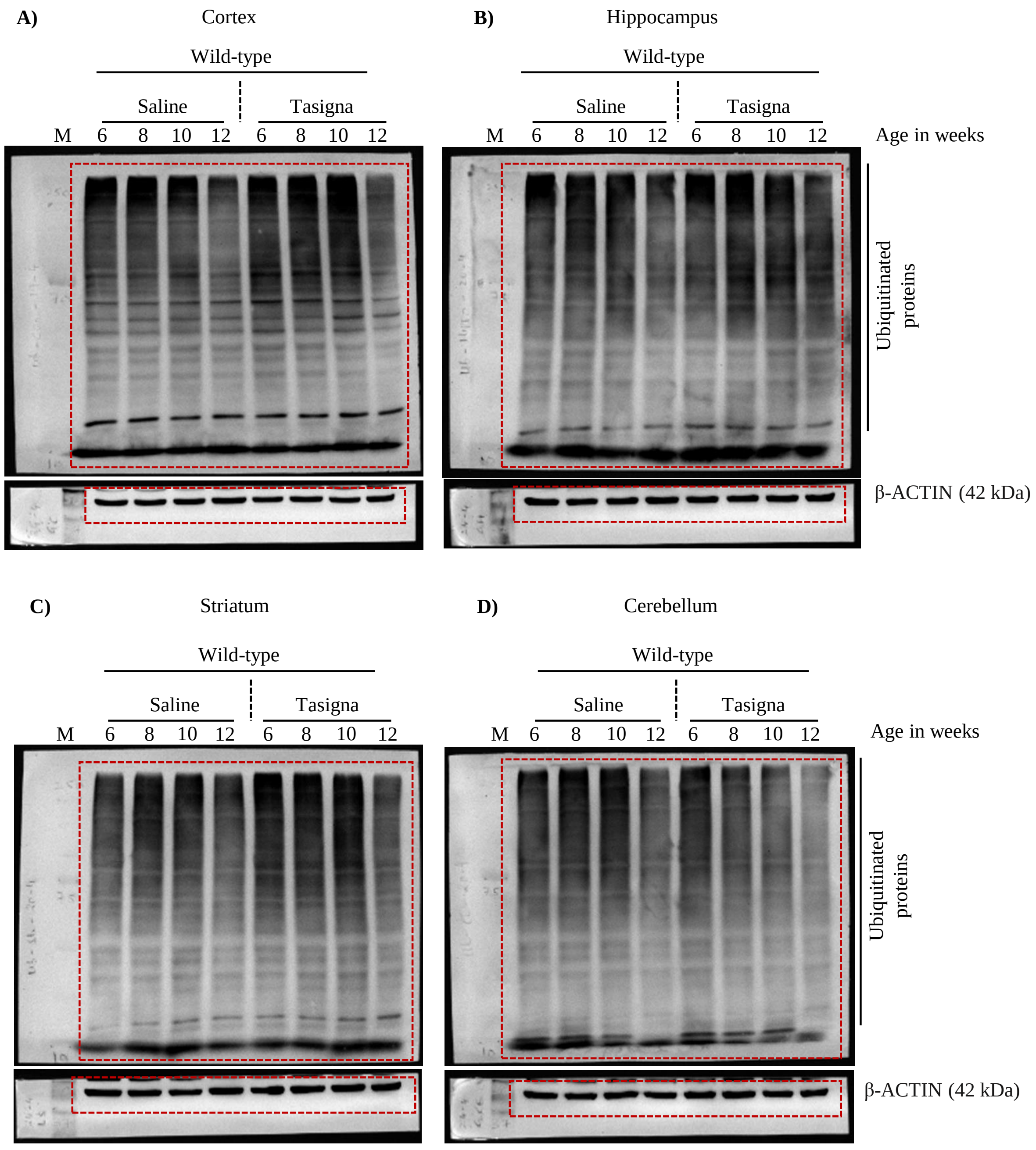

Supplementary figure S14
For figure S6

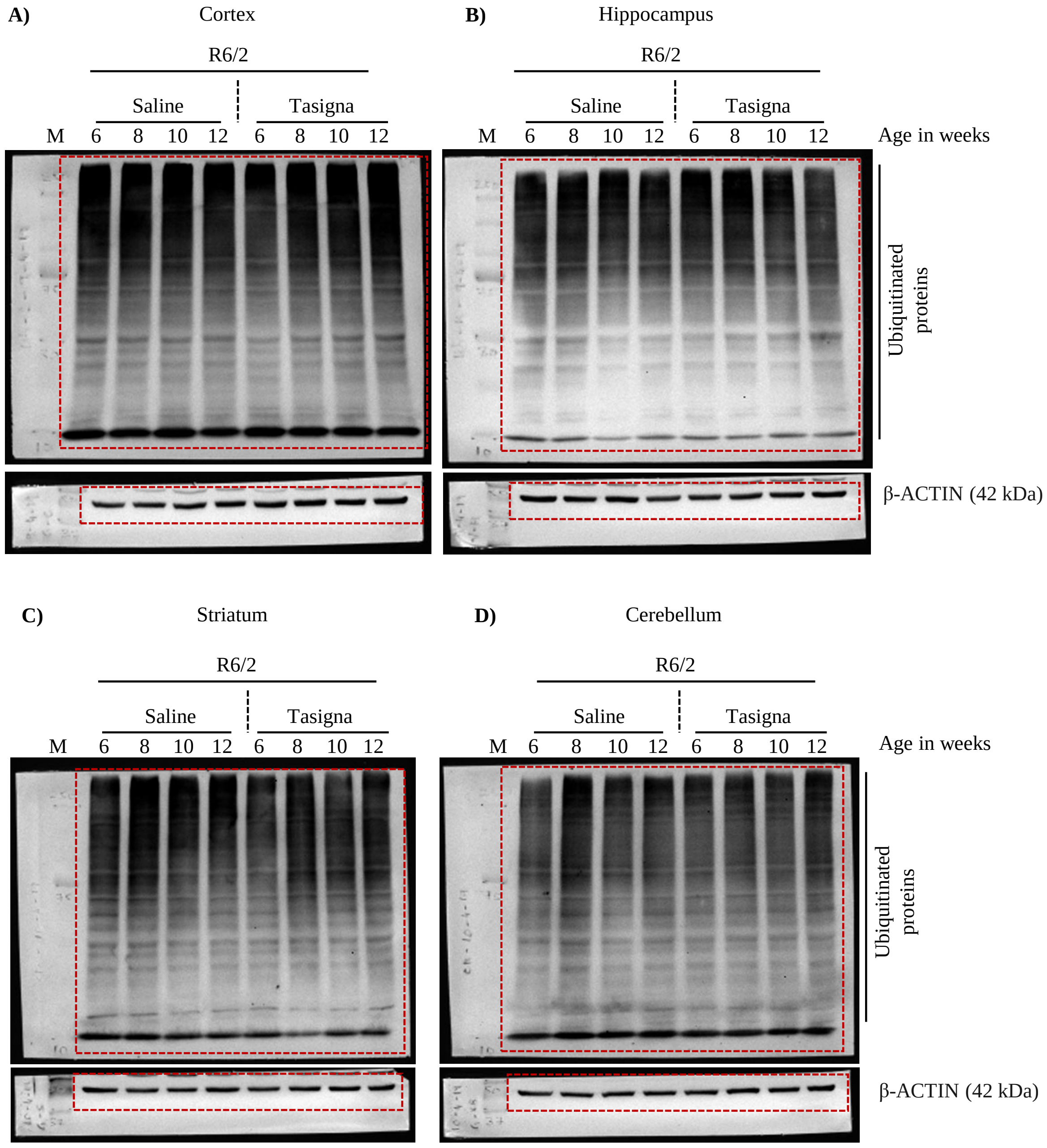

Supplementary figure S15
For figure S3

A)

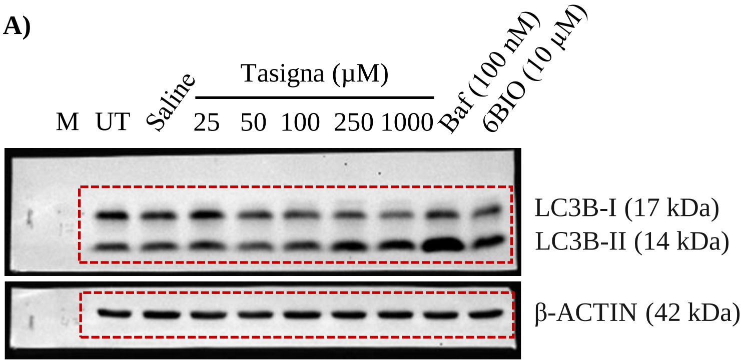

Supplementary Table 1

Figure S2. Ubiquitination is increased in the striatum at end-stage of disease progression in R6/2 - Stacking

Region	Genotype	Age (weeks)	Mean	±SEM
Cortex	Wild-type	2	13.70	2.03
	Wild-type	4	9.64	0.54
	Wild-type	8	7.71	0.44
	Wild-type	12	7.05	0.62
	R6/2	2	13.41	0.65
	R6/2	4	9.49	0.76
	R6/2	8	9.52	0.52
	R6/2	12	8.13	0.37
Hippocampus	Wild-type	2	11.08	0.94
	Wild-type	4	10.78	0.90
	Wild-type	8	9.76	1.21
	Wild-type	12	5.51	0.44
	R6/2	2	11.56	1.29
	R6/2	4	11.13	0.98
	R6/2	8	9.94	0.88
	R6/2	12	6.18	0.41
Striatum	Wild-type	2	9.50	0.44
	Wild-type	4	10.13	0.88
	Wild-type	8	11.58	1.04
	Wild-type	12	5.23	0.38
	R6/2	2	10.95	0.64
	R6/2	4	10.14	0.83
	R6/2	8	11.66	0.67
	R6/2	12	7.54	0.58
Cerebellum	Wild-type	2	11.13	1.18
	Wild-type	4	10.52	1.52
	Wild-type	8	9.99	0.81
	Wild-type	12	7.00	0.817
	R6/2	2	11.90	0.93
	R6/2	4	9.76	1.29
	R6/2	8	8.28	0.79
	R6/2	12	9.04	0.69

Supplementary Table 1

Figure S2. Ubiquitination is increased in the striatum at end-stage of disease progression in R6/2- Resolving

Region	Genotype	Age (weeks)	Mean	±SEM
**Cortex**	Wild-type	2	12.6	0.63
	Wild-type	4	9.63	0.62
	Wild-type	8	9.91	1.21
	Wild-type	12	11.49	0.37
	R6/2	2	10.88	0.91
	R6/2	4	10.64	1.05
	R6/2	8	11.74	1.08
	R6/2	12	12.80	1.03
**Hippocampus**	Wild-type	2	11.42	0.68
	Wild-type	4	9.92	0.72
	Wild-type	8	9.95	0.55
	Wild-type	12	13.02	0.69
	R6/2	2	11.31	1.22
	R6/2	4	10.72	0.40
	R6/2	8	11.09	0.74
	R6/2	12	12.83	0.97
**Striatum**	Wild-type	2	14.91	0.46
	Wild-type	4	10.84	0.72
	Wild-type	8	10.95	0.92
	Wild-type	12	10.82	0.56
	R6/2	2	15.44	0.88
	R6/2	4	11.18	1.06
	R6/2	8	11.48	0.78
	R6/2	12	13.40	0.79
**Cerebellum**	Wild-type	2	12.45	0.81
	Wild-type	4	10.04	0.34
	Wild-type	8	10.76	0.53
	Wild-type	12	11.80	1.07
	R6/2	2	13.13	0.73
	R6/2	4	9.44	0.82
	R6/2	8	8.72	1.05
	R6/2	12	12.83	1.22

Supplementary Table 2A: Cortex

Figure 2. Basal autophagy is not altered across different stages of disease progression in R6/2

ATG Proteins	Genotype	Age (weeks)	Mean	±SEM
**p62**	Wild-type	2	1.20	0.07
	Wild-type	4	1.47	0.15
	Wild-type	8	2.10	0.37
	Wild-type	12	1.40	0.07
	R6/2	2	1.25	0.11
	R6/2	4	1.27	0.10
	R6/2	8	1.92	0.31
	R6/2	12	1.28	0.09
**LC3B-I**	Wild-type	2	1.42	0.06
	Wild-type	4	0.99	0.09
	Wild-type	8	1.14	0.09
	Wild-type	12	1.29	0.12
	R6/2	2	1.34	0.10
	R6/2	4	0.93	0.05
	R6/2	8	1.19	0.06
	R6/2	12	0.91	0.06
**LC3B-II**	Wild-type	2	0.37	0.04
	Wild-type	4	0.20	0.01
	Wild-type	8	0.29	0.03
	Wild-type	12	0.36	0.05
	R6/2	2	0.33	0.06
	R6/2	4	0.19	0.01
	R6/2	8	0.26	0.02
	R6/2	12	0.34	0.06
**GABARAPL2**	Wild-type	2	1.62	0.15
	Wild-type	4	3.17	0.72
	Wild-type	8	1.73	0.17
	Wild-type	12	2.37	0.21
	R6/2	2	1.70	0.12
	R6/2	4	2.92	0.75
	R6/2	8	2.25	0.22
	R6/2	12	2.01	0.14

Supplementary Table 2B: Hippocampus

Figure 2. Basal autophagy is not altered across different stages of disease progression in R6/2

ATG Proteins	Genotype	Age (weeks)	Mean	±SEM
**p62**	Wild-type	2	1.51	0.07
	Wild-type	4	1.49	0.07
	Wild-type	8	2.41	0.37
	Wild-type	12	1.22	0.08
	R6/2	2	1.54	0.06
	R6/2	4	1.40	0.10
	R6/2	8	2.16	0.37
	R6/2	12	1.37	0.09
**LC3B-I**	Wild-type	2	1.49	0.10
	Wild-type	4	1.15	0.05
	Wild-type	8	1.59	0.31
	Wild-type	12	1.12	0.10
	R6/2	2	1.43	0.11
	R6/2	4	1.28	0.12
	R6/2	8	1.54	0.18
	R6/2	12	1.21	0.10
**LC3B-II**	Wild-type	2	0.39	0.04
	Wild-type	4	0.22	0.01
	Wild-type	8	0.32	0.05
	Wild-type	12	0.26	0.02
	R6/2	2	0.35	0.04
	R6/2	4	0.24	0.04
	R6/2	8	0.34	0.02
	R6/2	12	0.29	0.03
**GABARAPL2**	Wild-type	2	1.10	0.14
	Wild-type	4	1.94	0.21
	Wild-type	8	2.24	0.58
	Wild-type	12	1.20	0.10
	R6/2	2	1.11	0.19
	R6/2	4	1.59	0.20
	R6/2	8	2.06	0.25
	R6/2	12	1.28	0.07

Supplementary Table 2C: Striatum

Figure 2. Basal autophagy is not altered across different stages of disease progression in R6/2

ATG Proteins	Genotype	Age (weeks)	Mean	±SEM
**p62**	Wild-type	2	1.16	0.09
	Wild-type	4	1.41	0.06
	Wild-type	8	1.02	0.10
	Wild-type	12	1.23	0.12
	R6/2	2	1.22	0.06
	R6/2	4	1.23	0.04
	R6/2	8	1.01	0.08
	R6/2	12	1.52	0.15
**LC3B-I**	Wild-type	2	1.15	0.22
	Wild-type	4	1.66	0.14
	Wild-type	8	1.32	0.15
	Wild-type	12	1.17	0.11
	R6/2	2	1.14	0.17
	R6/2	4	1.49	0.09
	R6/2	8	1.22	0.05
	R6/2	12	1.11	0.10
**LC3B-II**	Wild-type	2	0.34	0.08
	Wild-type	4	0.46	0.06
	Wild-type	8	0.25	0.03
	Wild-type	12	0.34	0.04
	R6/2	2	0.33	0.05
	R6/2	4	0.43	0.06
	R6/2	8	0.27	0.02
	R6/2	12	0.40	0.05
**GABARAPL2**	Wild-type	2	1.40	0.19
	Wild-type	4	1.86	0.39
	Wild-type	8	1.03	0.10
	Wild-type	12	1.79	0.17
	R6/2	2	1.54	0.21
	R6/2	4	1.89	0.33
	R6/2	8	1.06	0.09
	R6/2	12	1.71	0.18

Supplementary Table 2D: Cerebellum

Figure 2: Basal autophagy is not altered across different stages of disease progression in R6/2

ATG Proteins	Genotype	Age (weeks)	Mean	±SEM
**p62**	Wild-type	2	0.92	0.15
	Wild-type	4	1.19	0.12
	Wild-type	8	1.88	0.20
	Wild-type	12	1.12	0.10
	R6/2	2	0.89	0.15
	R6/2	4	1.30	0.14
	R6/2	8	2.01	0.26
	R6/2	12	1.48	0.07
**LC3B-I**	Wild-type	2	1.36	0.19
	Wild-type	4	0.88	0.06
	Wild-type	8	1.51	0.19
	Wild-type	12	1.08	0.07
	R6/2	2	1.26	0.21
	R6/2	4	0.97	0.07
	R6/2	8	1.57	0.27
	R6/2	12	1.20	0.09
**LC3B-II**	Wild-type	2	0.52	0.06
	Wild-type	4	0.29	0.03
	Wild-type	8	0.41	0.07
	Wild-type	12	0.36	0.04
	R6/2	2	0.47	0.08
	R6/2	4	0.34	0.02
	R6/2	8	0.47	0.09
	R6/2	12	0.42	0.06
**GABARAPL2**	Wild-type	2	1.39	0.14
	Wild-type	4	1.35	0.10
	Wild-type	8	1.76	0.17
	Wild-type	12	1.74	0.16
	R6/2	2	1.33	0.24
	R6/2	4	1.61	0.11
	R6/2	8	1.88	0.12
	R6/2	12	1.71	0.16

Supplementary Table 3A: Bodyweight

Figure 4: Nilotinib (Tasigna^{TM}) is ineffective in rescuing the motor functions and improving the survival rate in R6/2

Behaviour	Genotype	Treatment	Age	Mean	±SEM
Body Weight	Wild-type	Saline	3	7.68988	0.2827
			4	10.6414	0.47108
			5	13.9945	0.72591
			6	17.7875	0.52892
			7	19.9922	0.64879
			8	20.9044	0.76691
			9	21.1268	0.76471
			10	21.8163	0.83004
			11	22.1125	1.17585
		Tasigna	3	7.56954	0.23735
			4	9.78294	0.35271
			5	12.2387	0.52157
			6	14.9314	0.63712
			7	17.4856	0.38347
			8	19.5216	0.30248
			9	20.3803	0.25805
			10	21.4999	0.26601
			11	22.419	0.26108
	R6/2	Saline	3	6.1805	0.23866
			4	8.23017	0.34454
			5	12.0216	0.67227
			6	14.4976	0.77895
			7	14.6814	0.70505
			8	16.1852	0.49539
			9	17.8888	0.46958
			10	17.8467	0.43305
			11	17.4274	0.46263
		Tasigna	3	5.93342	0.24898
			4	7.83225	0.46599
			5	10.8631	0.71026
			6	13.1414	0.87308
			7	13.602	0.88234
			8	14.9017	0.80569
			9	15.5084	0.79903
			10	16.1381	0.84886
			11	15.3731	0.90494

Supplementary Table 3B: Open-Field test

Figure 4: Nilotinib (Tasigna^{TM}) is ineffective in rescuing the motor functions and improving the survival rate in R6/2

Behaviour	Genotype	Treatment	Age	Mean	±SEM
**Open-Field Test**	Wild-type	Saline	6	976.5	153.4
			7	640.8	128.3
			8	438.5	114
			9	388.2	101.5
			10	354.5	97.94
			11	287.6	98.71
		Tasigna	6	1190	202.5
			7	806.1	98.18
			8	640	115.8
			9	514.4	150.6
			10	411.7	140.4
			11	399.3	63.72
	R6/2	Saline	6	649.1	124
			7	525.7	137.8
			8	355.4	101.8
			9	541.4	87.99
			10	679.7	155.1
			11	541.1	229
		Tasigna	6	878.7	63.57
			7	732.6	140.9
			8	496.3	86.32
			9	369.5	60.04
			10	299.7	76.63
			11	439.4	151.5

Supplementary Table 3C: Rotarod test

Figure 4: Nilotinib (Tasigna^{TM}) is ineffective in rescuing the motor functions and improving the survival rate in R6/2

Behaviour	Genotype	Treatment	Age	Mean	±SEM
Rotarod Test	Wild-type	Saline	6	44.5	3.971
			7	34.7	4.714
			8	29.6	5.09
			9	41.7	6.254
			10	43	6.098
			11	47.1	3.125
		Tasigna	6	49	4.802
			7	36.7	6.782
			8	44.2	6.074
			9	46	6.122
			10	52.3	5.055
			11	49.8	4.369
	R6/2	Saline	6	29	6.736
			7	20.6	6.816
			8	14.3	5.663
			9	6.06	3.77
			10	2.56	1.467
			11	0.78	0.719
		Tasigna	6	28	6.704
			7	25.7	6.561
			8	10.4	3.347
			9	4.5	2.53
			10	1.06	0.567
			11	1.14	1.139

Supplementary Table 3D: Hind-limb clasping test

Figure 4: Nilotinib (Tasigna^{TM}) is ineffective in rescuing the motor functions and improving the survival rate in R6/2

Behaviour	Genotype	Treatment	Age	Mean	±SEM
Hind-limb clasping Test	Wild-type	Saline	6	0	0
			7	0	0
			8	0	0
			9	0	0
			10	0	0
			11	0	0
		Tasigna	6	0	0
			7	0	0
			8	0	0
			9	0	0
			10	0	0
			11	0	0
	R6/2	Saline	6	0.58	0.358
			7	1.17	0.345
			8	2	0.64
			9	1.83	0.548
			10	3.08	0.57
			11	3.08	0.596
		Tasigna	6	0.92	0.452
			7	0.92	0.452
			8	1.75	0.494
			9	2.33	0.527
			10	2.5	0.435
			11	3.67	0.466

Supplementary Table 4A: Cortex

Figure 5: Nilotinib (Tasigna^{TM}) has no effect in inducing autophagy in wild-type control mice

ATG Proteins	Genotype	Treatment	Age (weeks)	Mean	±SEM
**p62**	Wild-type	Saline	6	1.05	0.09
	Wild-type	Saline	8	1.25	0.20
	Wild-type	Saline	10	1.11	0.13
	Wild-type	Saline	12	0.78	0.03
	Wild-type	Tasigna	6	0.98	0.05
	Wild-type	Tasigna	8	1.57	0.23
	Wild-type	Tasigna	10	1.08	0.06
	Wild-type	Tasigna	12	1.16	0.12
**LC3B-I**	Wild-type	Saline	6	1.43	0.17
	Wild-type	Saline	8	1.67	0.27
	Wild-type	Saline	10	1.46	0.24
	Wild-type	Saline	12	1.11	0.03
	Wild-type	Tasigna	6	1.24	0.13
	Wild-type	Tasigna	8	1.61	0.36
	Wild-type	Tasigna	10	1.42	0.15
	Wild-type	Tasigna	12	1.25	0.20
**LC3B-II**	Wild-type	Saline	6	0.25	0.05
	Wild-type	Saline	8	1.00	0.27
	Wild-type	Saline	10	0.93	0.16
	Wild-type	Saline	12	0.18	0.03
	Wild-type	Tasigna	6	0.22	0.04
	Wild-type	Tasigna	8	0.67	0.11
	Wild-type	Tasigna	10	0.92	0.07
	Wild-type	Tasigna	12	0.30	0.04
**GABARAPL2**	Wild-type	Saline	6	1.33	0.14
	Wild-type	Saline	8	1.46	0.08
	Wild-type	Saline	10	1.25	0.26
	Wild-type	Saline	12	1.14	0.09
	Wild-type	Tasigna	6	1.47	0.09
	Wild-type	Tasigna	8	1.50	0.12
	Wild-type	Tasigna	10	1.90	0.15
	Wild-type	Tasigna	12	1.36	0.15

Supplementary Table 4B: Hippocampus

Figure 5: Nilotinib (Tasigna^{TM}) has no effect in inducing autophagy in wild-type control mice

ATG Proteins	Genotype	Treatment	Age (weeks)	Mean	±SEM
**p62**	Wild-type	Saline	6	0.71	0.12
	Wild-type	Saline	8	1.44	0.21
	Wild-type	Saline	10	1.35	0.17
	Wild-type	Saline	12	0.78	0.10
	Wild-type	Tasigna	6	1.05	0.07
	Wild-type	Tasigna	8	1.33	0.24
	Wild-type	Tasigna	10	1.02	0.08
	Wild-type	Tasigna	12	0.76	0.02
**LC3B-I**	Wild-type	Saline	6	1.08	0.08
	Wild-type	Saline	8	1.55	0.21
	Wild-type	Saline	10	1.52	0.21
	Wild-type	Saline	12	0.97	0.02
	Wild-type	Tasigna	6	1.34	0.19
	Wild-type	Tasigna	8	1.56	0.40
	Wild-type	Tasigna	10	1.16	0.18
	Wild-type	Tasigna	12	1.09	0.04
**LC3B-II**	Wild-type	Saline	6	0.28	0.04
	Wild-type	Saline	8	0.85	0.17
	Wild-type	Saline	10	1.00	0.10
	Wild-type	Saline	12	0.18	0.02
	Wild-type	Tasigna	6	0.29	0.05
	Wild-type	Tasigna	8	0.74	0.21
	Wild-type	Tasigna	10	0.83	0.02
	Wild-type	Tasigna	12	0.21	0.04
**GABARAPL2**	Wild-type	Saline	6	1.17	0.06
	Wild-type	Saline	8	1.71	0.34
	Wild-type	Saline	10	1.50	0.30
	Wild-type	Saline	12	0.89	0.03
	Wild-type	Tasigna	6	1.10	0.02
	Wild-type	Tasigna	8	1.33	0.16
	Wild-type	Tasigna	10	1.23	0.29
	Wild-type	Tasigna	12	1.08	0.02

Supplementary Table 4C: Striatum

Figure 5: Nilotinib (Tasigna^{TM}) has no effect in inducing autophagy in wild-type control mice

ATG Proteins	Genotype	Treatment	Age (weeks)	Mean	±SEM
**p62**	Wild-type	Saline	6	1.03	0.08
	Wild-type	Saline	8	0.85	0.13
	Wild-type	Saline	10	1.20	0.16
	Wild-type	Saline	12	1.23	0.09
	Wild-type	Tasigna	6	1.23	0.19
	Wild-type	Tasigna	8	0.89	0.12
	Wild-type	Tasigna	10	0.94	0.05
	Wild-type	Tasigna	12	1.21	0.11
**LC3B-I**	Wild-type	Saline	6	1.32	0.24
	Wild-type	Saline	8	0.97	0.08
	Wild-type	Saline	10	1.12	0.24
	Wild-type	Saline	12	1.48	0.20
	Wild-type	Tasigna	6	1.76	0.28
	Wild-type	Tasigna	8	0.85	0.10
	Wild-type	Tasigna	10	0.84	0.08
	Wild-type	Tasigna	12	1.40	0.14
**LC3B-II**	Wild-type	Saline	6	0.62	0.10
	Wild-type	Saline	8	0.16	0.01
	Wild-type	Saline	10	0.20	0.03
	Wild-type	Saline	12	0.62	0.07
	Wild-type	Tasigna	6	0.78	0.16
	Wild-type	Tasigna	8	0.14	0.01
	Wild-type	Tasigna	10	0.16	0.01
	Wild-type	Tasigna	12	0.57	0.05
**GABARAPL2**	Wild-type	Saline	6	1.28	0.14
	Wild-type	Saline	8	1.04	0.04
	Wild-type	Saline	10	1.08	0.13
	Wild-type	Saline	12	0.23	0.03
	Wild-type	Tasigna	6	1.32	0.13
	Wild-type	Tasigna	8	0.99	0.06
	Wild-type	Tasigna	10	1.08	0.06
	Wild-type	Tasigna	12	0.26	0.01

Supplementary Table 4D: Cerebellum

Figure 5: Nilotinib (Tasigna^{TM}) has no effect in inducing autophagy in wild-type control mice

ATG Proteins	Genotype	Treatment	Age (weeks)	Mean	±SEM
**p62**	Wild-type	Saline	6	0.83	0.07
	Wild-type	Saline	8	1.41	0.21
	Wild-type	Saline	10	0.91	0.07
	Wild-type	Saline	12	1.05	0.13
	Wild-type	Tasigna	6	1.02	0.08
	Wild-type	Tasigna	8	0.88	0.06
	Wild-type	Tasigna	10	0.87	0.05
	Wild-type	Tasigna	12	1.09	0.19
**LC3B-I**	Wild-type	Saline	6	1.22	0.21
	Wild-type	Saline	8	1.47	0.14
	Wild-type	Saline	10	0.88	0.02
	Wild-type	Saline	12	1.19	0.18
	Wild-type	Tasigna	6	1.35	0.18
	Wild-type	Tasigna	8	0.97	0.03
	Wild-type	Tasigna	10	0.92	0.04
	Wild-type	Tasigna	12	1.65	0.38
**LC3B-II**	Wild-type	Saline	6	0.51	0.20
	Wild-type	Saline	8	0.33	0.06
	Wild-type	Saline	10	0.17	0.01
	Wild-type	Saline	12	0.35	0.12
	Wild-type	Tasigna	6	0.60	0.21
	Wild-type	Tasigna	8	0.14	0.02
	Wild-type	Tasigna	10	0.17	0.01
	Wild-type	Tasigna	12	0.71	0.15
**GABARAPL2**	Wild-type	Saline	6	1.43	0.10
	Wild-type	Saline	8	1.80	0.08
	Wild-type	Saline	10	1.43	0.07
	Wild-type	Saline	12	1.50	0.15
	Wild-type	Tasigna	6	1.71	0.19
	Wild-type	Tasigna	8	1.39	0.15
	Wild-type	Tasigna	10	1.64	0.16
	Wild-type	Tasigna	12	1.50	0.06

Supplementary Table 5A: Cortex

Figure 6: Nilotinib (Tasigna^{TM}) is ineffective in inducing autophagy across different stages of disease progression in R6/2

ATG Proteins	Genotype	Treatment	Age (weeks)	Mean	±SEM
**p62**	R6/2	Saline	6	1.23	0.11
	R6/2	Saline	8	1.31	0.09
	R6/2	Saline	10	1.32	0.03
	R6/2	Saline	12	1.04	0.09
	R6/2	Tasigna	6	1.48	0.15
	R6/2	Tasigna	8	1.06	0.05
	R6/2	Tasigna	10	1.26	0.06
	R6/2	Tasigna	12	1.02	0.01
**LC3B-I**	R6/2	Saline	6	1.21	0.05
	R6/2	Saline	8	1.33	0.16
	R6/2	Saline	10	1.41	0.10
	R6/2	Saline	12	1.26	0.08
	R6/2	Tasigna	6	1.56	0.07
	R6/2	Tasigna	8	1.16	0.02
	R6/2	Tasigna	10	1.25	0.05
	R6/2	Tasigna	12	1.35	0.15
**LC3B-II**	R6/2	Saline	6	0.26	0.09
	R6/2	Saline	8	0.32	0.09
	R6/2	Saline	10	0.36	0.06
	R6/2	Saline	12	0.21	0.03
	R6/2	Tasigna	6	0.41	0.12
	R6/2	Tasigna	8	0.28	0.04
	R6/2	Tasigna	10	0.30	0.02
	R6/2	Tasigna	12	0.21	0.04
**GABARAPL2**	R6/2	Saline	6	1.19	0.19
	R6/2	Saline	8	1.57	0.41
	R6/2	Saline	10	1.66	0.41
	R6/2	Saline	12	0.91	0.19
	R6/2	Tasigna	6	1.43	0.31
	R6/2	Tasigna	8	1.29	0.35
	R6/2	Tasigna	10	1.38	0.36
	R6/2	Tasigna	12	1.09	0.27

Supplementary Table 5B: Hippocampus

Figure 6: Nilotinib (Tasigna^{TM}) is ineffective in inducing autophagy across different stages of disease progression in R6/2

ATG Proteins	Genotype	Treatment	Age (weeks)	Mean	±SEM
**p62**	R6/2	Saline	6	1.34	0.49
	R6/2	Saline	8	1.05	0.09
	R6/2	Saline	10	1.17	0.20
	R6/2	Saline	12	0.84	0.09
	R6/2	Tasigna	6	1.13	0.24
	R6/2	Tasigna	8	1.23	0.06
	R6/2	Tasigna	10	1.01	0.06
	R6/2	Tasigna	12	0.76	0.08
**LC3B-I**	R6/2	Saline	6	1.21	0.29
	R6/2	Saline	8	1.09	0.18
	R6/2	Saline	10	1.30	0.35
	R6/2	Saline	12	1.06	0.11
	R6/2	Tasigna	6	1.17	0.32
	R6/2	Tasigna	8	1.14	0.19
	R6/2	Tasigna	10	1.05	0.18
	R6/2	Tasigna	12	1.05	0.18
**LC3B-II**	R6/2	Saline	6	0.34	0.05
	R6/2	Saline	8	0.37	0.09
	R6/2	Saline	10	0.42	0.13
	R6/2	Saline	12	0.34	0.04
	R6/2	Tasigna	6	0.29	0.04
	R6/2	Tasigna	8	0.34	0.09
	R6/2	Tasigna	10	0.32	0.07
	R6/2	Tasigna	12	0.26	0.05
**GABARAPL2**	R6/2	Saline	6	1.86	0.37
	R6/2	Saline	8	3.16	0.91
	R6/2	Saline	10	2.90	0.86
	R6/2	Saline	12	2.39	0.42
	R6/2	Tasigna	6	1.73	0.06
	R6/2	Tasigna	8	2.60	0.60
	R6/2	Tasigna	10	2.84	0.57
	R6/2	Tasigna	12	2.21	0.41

Supplementary Table 5C: Striatum

Figure 6: Nilotinib (Tasigna^{TM}) is ineffective in inducing autophagy across different stages of disease progression in R6/2

ATG Proteins	Genotype	Treatment	Age (weeks)	Mean	±SEM
**p62**	R6/2	Saline	6	1.25	0.18
	R6/2	Saline	8	1.41	0.14
	R6/2	Saline	10	1.10	0.14
	R6/2	Saline	12	1.05	0.05
	R6/2	Tasigna	6	1.36	0.23
	R6/2	Tasigna	8	1.22	0.11
	R6/2	Tasigna	10	1.18	0.03
	R6/2	Tasigna	12	1.02	0.15
**LC3B-I**	R6/2	Saline	6	1.03	0.38
	R6/2	Saline	8	1.06	0.07
	R6/2	Saline	10	0.86	0.02
	R6/2	Saline	12	1.57	0.27
	R6/2	Tasigna	6	1.46	0.50
	R6/2	Tasigna	8	0.83	0.05
	R6/2	Tasigna	10	1.00	0.17
	R6/2	Tasigna	12	1.55	0.29
**LC3B-II**	R6/2	Saline	6	0.22	0.06
	R6/2	Saline	8	0.30	0.04
	R6/2	Saline	10	0.26	0.03
	R6/2	Saline	12	0.32	0.02
	R6/2	Tasigna	6	0.40	0.08
	R6/2	Tasigna	8	0.21	0.01
	R6/2	Tasigna	10	0.33	0.07
	R6/2	Tasigna	12	0.33	0.03
**GABARAPL2**	R6/2	Saline	6	1.51	0.17
	R6/2	Saline	8	3.04	0.58
	R6/2	Saline	10	2.12	0.15
	R6/2	Saline	12	1.65	0.27
	R6/2	Tasigna	6	2.19	0.12
	R6/2	Tasigna	8	2.25	0.27
	R6/2	Tasigna	10	2.85	0.53
	R6/2	Tasigna	12	1.78	0.06

Supplementary Table 5D: Cerebellum

Figure 6: Nilotinib (Tasigna^{TM}) is ineffective in inducing autophagy across different stages of disease progression in R6/2

ATG Proteins	Genotype	Treatment	Age (weeks)	Mean	±SEM
**p62**	R6/2	Saline	6	1.10	0.51
	R6/2	Saline	8	1.20	0.36
	R6/2	Saline	10	1.04	0.27
	R6/2	Saline	12	0.71	0.13
	R6/2	Tasigna	6	1.12	0.41
	R6/2	Tasigna	8	1.07	0.19
	R6/2	Tasigna	10	1.09	0.11
	R6/2	Tasigna	12	1.01	0.04
**LC3B-I**	R6/2	Saline	6	1.74	0.63
	R6/2	Saline	8	1.32	0.31
	R6/2	Saline	10	1.07	0.20
	R6/2	Saline	12	1.28	0.25
	R6/2	Tasigna	6	1.46	0.40
	R6/2	Tasigna	8	1.13	0.26
	R6/2	Tasigna	10	0.98	0.02
	R6/2	Tasigna	12	1.55	0.27
**LC3B-II**	R6/2	Saline	6	0.39	0.14
	R6/2	Saline	8	0.44	0.19
	R6/2	Saline	10	0.34	0.12
	R6/2	Saline	12	0.43	0.16
	R6/2	Tasigna	6	0.37	0.12
	R6/2	Tasigna	8	0.38	0.15
	R6/2	Tasigna	10	0.32	0.09
	R6/2	Tasigna	12	0.51	0.18
**GABARAPL2**	R6/2	Saline	6	1.60	0.14
	R6/2	Saline	8	1.81	0.02
	R6/2	Saline	10	1.70	0.14
	R6/2	Saline	12	1.96	0.25
	R6/2	Tasigna	6	1.86	0.05
	R6/2	Tasigna	8	1.51	0.13
	R6/2	Tasigna	10	1.53	0.09
	R6/2	Tasigna	12	3.08	0.77

Supplementary Table 6

Figure 7: Nilotinib (Tasigna^{TM}) is ineffective in enhancing the clearance of mHTT aggregates at any given stage of disease progression in R6/2

Region	Genotype	Treatment	Age (weeks)	Mean	±SEM
**Cortex**	R6/2	Saline	6	8.56	2.02
	R6/2	Saline	8	8.14	1.75
	R6/2	Saline	10	9.59	0.81
	R6/2	Saline	12	10.74	1.19
	R6/2	Tasigna	6	12.4	1.62
	R6/2	Tasigna	8	12.08	1.48
	R6/2	Tasigna	10	13.30	1.83
	R6/2	Tasigna	12	11.92	2.25
**Hippocampus**	R6/2	Saline	6	10.42	3.61
	R6/2	Saline	8	9.73	2.14
	R6/2	Saline	10	10.30	0.77
	R6/2	Saline	12	10.11	1.37
	R6/2	Tasigna	6	9.19	2.50
	R6/2	Tasigna	8	11.21	1.53
	R6/2	Tasigna	10	13.01	1.29
	R6/2	Tasigna	12	13.72	1.25
**Striatum**	R6/2	Saline	6	10.45	0.44
	R6/2	Saline	8	10.09	0.46
	R6/2	Saline	10	12.25	1.10
	R6/2	Saline	12	14.75	1.66
	R6/2	Tasigna	6	13.98	1.33
	R6/2	Tasigna	8	13.78	0.70
	R6/2	Tasigna	10	14.70	1.54
	R6/2	Tasigna	12	14.95	1.18
**Cerebellum**	R6/2	Saline	6	10.73	0.23
	R6/2	Saline	8	11.60	1.97
	R6/2	Saline	10	13.47	0.84
	R6/2	Saline	12	13.93	0.70
	R6/2	Tasigna	6	15.26	0.51
	R6/2	Tasigna	8	15.11	0.59
	R6/2	Tasigna	10	13.29	0.95
	R6/2	Tasigna	12	12.49	0.89

Supplementary Table 7

Figure S4: Nilotinib (Tasigna^{TM}) has no effect on UBIQUITIN profile of the cell in wild-type control mice

Region	Genotype	Treatment	Age (weeks)	Mean	±SEM
**Cortex**	Wild-type	Saline	6	16.07	1.90
	Wild-type	Saline	8	23.13	4.86
	Wild-type	Saline	10	17.55	2.24
	Wild-type	Saline	12	11.97	0.90
	Wild-type	Tasigna	6	14.75	0.53
	Wild-type	Tasigna	8	17.95	3.26
	Wild-type	Tasigna	10	15.20	1.14
	Wild-type	Tasigna	12	15.47	3.42
**Hippocampus**	Wild-type	Saline	6	10.19	0.71
	Wild-type	Saline	8	17.00	1.68
	Wild-type	Saline	10	13.91	1.82
	Wild-type	Saline	12	10.80	1.43
	Wild-type	Tasigna	6	11.27	1.77
	Wild-type	Tasigna	8	11.67	1.59
	Wild-type	Tasigna	10	10.03	1.03
	Wild-type	Tasigna	12	13.32	0.44
**Striatum**	Wild-type	Saline	6	14.01	2.61
	Wild-type	Saline	8	7.56	1.06
	Wild-type	Saline	10	10.36	2.64
	Wild-type	Saline	12	15.82	2.48
	Wild-type	Tasigna	6	15.14	2.13
	Wild-type	Tasigna	8	6.84	1.72
	Wild-type	Tasigna	10	11.34	2.19
	Wild-type	Tasigna	12	13.09	0.78
**Cerebellum**	Wild-type	Saline	6	13.81	1.07
	Wild-type	Saline	8	15.78	1.37
	Wild-type	Saline	10	10.88	1.03
	Wild-type	Saline	12	9.95	0.82
	Wild-type	Tasigna	6	13.23	0.83
	Wild-type	Tasigna	8	9.29	1.25
	Wild-type	Tasigna	10	10.89	0.51
	Wild-type	Tasigna	12	10.67	1.45

Supplementary Table 8

Figure S5. Nilotinib (Tasigna^{TM}) has no effect on UBIQUITIN profile of the cell across different stages of disease progression in R6/2

Region	Genotype	Treatment	Age (weeks)	Mean	±SEM
**Cortex**	R6/2	Saline	6	14.57	2.18
	R6/2	Saline	8	14.74	1.82
	R6/2	Saline	10	12.14	2.50
	R6/2	Saline	12	14.03	1.46
	R6/2	Tasigna	6	14.95	1.24
	R6/2	Tasigna	8	9.79	3.06
	R6/2	Tasigna	10	12.74	2.62
	R6/2	Tasigna	12	13.21	1.03
**Hippocampus**	R6/2	Saline	6	17.94	5.68
	R6/2	Saline	8	13.55	1.27
	R6/2	Saline	10	13.39	1.72
	R6/2	Saline	12	11.42	0.61
	R6/2	Tasigna	6	11.78	2.70
	R6/2	Tasigna	8	12.09	1.85
	R6/2	Tasigna	10	12.68	1.68
	R6/2	Tasigna	12	10.67	1.24
**Striatum**	R6/2	Saline	6	12.21	0.59
	R6/2	Saline	8	14.24	1.43
	R6/2	Saline	10	11.93	0.70
	R6/2	Saline	12	12.57	2.02
	R6/2	Tasigna	6	12.00	1.13
	R6/2	Tasigna	8	9.58	1.91
	R6/2	Tasigna	10	11.58	0.29
	R6/2	Tasigna	12	11.74	0.67
**Cerebellum**	R6/2	Saline	6	7.01	1.46
	R6/2	Saline	8	13.88	2.10
	R6/2	Saline	10	11.12	0.78
	R6/2	Saline	12	7.74	1.48
	R6/2	Tasigna	6	6.93	2.14
	R6/2	Tasigna	8	11.01	0.83
	R6/2	Tasigna	10	11.01	0.32
	R6/2	Tasigna	12	8.82	1.70

Supplementary Table 9

Figure S3. Nilotinib (Tasigna™) induces autophagy in HeLa cells in dosage-dependent manner.

Treatment	Mean	SEM
UT	1.09253	0.14141
Saline	1	0.08171
Tasigna (25µM)	1.34689	0.17185
Tasigna (50µM)	1.45945	0.20051
Tasigna (100µM)	1.77652	0.19312
Tasigna (250µM)	2.13098	0.22358
Tasigna (1mM)	3.09051	0.38477
Bafilomycin A1 (100nM)	2.85307	0.33123
6BIO (10mM)	2.0218	0.09025

Supplementary figures

Figure S1. Dynamics of mHTT aggregates formation in R6/2 across different stages of disease progression in R6/2.

A. Representative ponceau S stained blot for mHTT aggregates for Cortex (C). **B, C, D.** Blots for mHTT aggregates with different exposures for the region Cortex (C) from 2, 4, 8, and 12 weeks. **E.** Representative ponceau S stained blot for mHTT aggregates for Hippocampus (H). **F, G, H.** Blots for mHTT aggregates with different exposures for the region Hippocampus (H) from 2, 4, 8, and 12 weeks. **I.** Representative ponceau S stained blot for mHTT aggregates for Striatum (S). **J, K, L.** Blots for mHTT aggregates with different exposures for the region Striatum (S) from 2, 4, 8, and 12 weeks. **M.** Representative ponceau S stained blot for mHTT aggregates for Cerebellum (CB). **N, O, P.** Blots for mHTT aggregates with different exposures for the region Cerebellum (CB) from 2, 4, 8, and 12 weeks. N=5 for 2 weeks, N=7 for 4 and 8 weeks, and N=12 for 12 weeks for both the genotypes wild-type and R6/2. N=number of mice, Stacking and resolving are separated by a thick vertical line. The genotypes are separated by vertical dashed lines. kDa – Molecular weight in kiloDaltons.

Figure S2. Ubiquitination is increased in the striatum at end-stage of disease progression in R6/2

A, B, C and D. Representative immunoblot for UBIQUITIN profile from 2, 4, 8, and 12 weeks in the cortex, hippocampus, striatum and cerebellum, respectively. **E – H.** Representative ponceau S stained blot for UBIQUITIN profile from 2, 4, 8, and 12 weeks in the cortex, Hippocampus, striatum and cerebellum, respectively. **I – L.** Quantified bar graphs for UBIQUITIN profile (Stacking) for cortex: Age x Genotype ($F_{(3,54)}$=0.82, p=0.48), hippocampus: Age x Genotype ($F_{(3,54)}$=0.04, p=0.98), striatum: Age x Genotype ($F_{(3,54)}$=1.49, p=0.22), and cerebellum: Age x Genotype ($F_{(3,54)}$=1.57, p=0.20), respectively. **M – P.** Quantified bar graphs for UBIQUITIN profile (Resolving) for cortex: Age x Genotype ($F_{(3,54)}$=1.095, p=0.35), hippocampus: Age x Genotype ($F_{(3,54)}$=0.31, p=0.81), striatum: Age x Genotype ($F_{(3,54)}$=1.08, p=0.36), and cerebellum: Age x Genotype ($F_{(3,54)}$=0.89, p=0.44), respectively. N=5 for 2 weeks, N=7 for 4 and 8 weeks, and N=12 for 12 weeks for both the genotypes wild-type and R6/2. Error bars indicate ±SEM. (Mean and ±SEM values are represented in supplementary **table 1**). N=number of mice. The genotypes are separated by vertical dashed lines. Statistical analysis was done by Two-way ANOVA followed by Bonferroni *post-hoc* test. Image brightness and contrast were adjusted for representative purpose. Full uncropped raw blots are represented in the supplementary figure S9.

Figure S3. Nilotinib (Tasigna^{TM}) induces autophagy in HeLa cells in a dosage-dependent manner.

A. Representative western blot showing an increase in the expression of ATG protein LC3B upon treatment with Nilotinib (Tasigna^{TM}). β-ACTIN is used as a loading control. **B.** Quantified bar graphs showing induction of autophagy (ratio of LC3B-II/LC3B-I) in a dosage-dependent manner. ($F_{(8,18)}$ =10.91, p<0.0001). Bafilomycin (100 nM), and 6BIO (10 µM) were used as negative and positive controls of autophagy, respectively. n=3. Error bars indicate ±SEM. (Mean and ±SEM values are represented in the supplementary **table)**. n=number of biological replicates. Statistical analysis was done by One-way ANOVA followed by Bonferroni *post-hoc* test. Full uncropped raw blots are represented in the supplementary figure S15.

Figure S4. Nilotinib (Tasigna^{TM}) is ineffective in enhancing the clearance of mHTT aggregates at any given stage of disease progression in R6/2.

A. Representative ponceau S stained blot for mHTT aggregates for Cortex. **B, C, D.** Blots for mHTT aggregates with different exposures for the region Cortex (C) at 6, 8, 10, and 12 weeks from Saline and Tasigna treated R6/2 mice. **E.** Representative ponceau S stained blot for mHTT aggregates for Hippocampus. **F, G, H.** Blots for mHTT aggregates with different exposures for the region Hippocampus at 6, 8, 10, and 12 weeks from Saline and Tasigna treated R6/2 mice. **I.** Representative ponceau S stained blot for mHTT aggregates for Striatum. **J, K, L.** Blots for mHTT aggregates with different exposures for the region Striatum at 6, 8, 10, and 12 weeks from Saline and Tasigna treated R6/2 mice. **M.** Representative ponceau S stained blot for mHTT aggregates for Cerebellum. **N, O, P.** Blots for mHTT aggregates with different exposures for the region Cerebellum at 6, 8, 10, and 12 weeks from Saline and Tasigna treated R6/2 mice. N = 3 (R6/2-Saline), and (R6/2-Tasigna) for all the 4 age groups 6, 8, 10, and 12 weeks. N=number of mice. Stacking and resolving are separated by a thick vertical line. The treatment groups are separated by vertical dashed lines. kDa – Molecular weight in kiloDaltons.

Figure S5. Nilotinib (Tasigna^{TM}) has no effect on UBIQUITIN profile of the cell in wild-type control mice.

A, B, C and D. Representative immunoblot for UBIQUITIN profile from 6, 8, 10, and 12 weeks in the cortex, hippocampus, striatum and cerebellum, respectively. **E – H.** Summary of quantified bar graphs for UBIQUITIN profile for cortex: Age x treatment ($F_{(3,12)}$=2.24, p=0.13), hippocampus: Age x treatment ($F_{(3,12)}$=5.70, p=0.01), striatum: Age x treatment ($F_{(3,12)}$=0.31, p=0.81), and cerebellum: Age x treatment ($F_{(3,12)}$=4.48, p=0.02), respectively. N = 3 (WT-Saline), and (WT-Tasigna) for all the 4 age groups 6, 8, 10, and 12 weeks. Error bars indicate ±SEM. (Mean and ±SEM values are represented in supplementary **table 7**). N=number of mice. The treatment groups are separated by vertical dashed lines. Statistical analysis was done by Two-way ANOVA followed by Bonferroni *post-hoc* test. Image brightness and contrast were adjusted for representative purpose. Full uncropped raw blots are represented in the supplementary figure S13.

Figure S6. Nilotinib (Tasigna^{TM}) does not affect the UBIQUITIN profile of the cell across different stages of disease progression in R6/2.

A, B, C and D. Representative immunoblot for UBIQUITIN profile from 6, 8, 10, and 12 weeks in the cortex, hippocampus, striatum and cerebellum, respectively. **E – H.** Summary of quantified bar graphs for UBIQUITIN profile for cortex: Age x treatment ($F_{(3,12)}$=2.40, p=0.11), hippocampus: Age x treatment ($F_{(3,12)}$=0.58, p=0.63), striatum: Age x treatment ($F_{(3,12)}$=1.23, p=0.3428), and cerebellum: Age x treatment ($F_{(3,12)}$=0.62, p=0.61) respectively. N = 3 (R6/2-Saline), and (R6/2-Tasigna) for all the 4 age groups 6, 8, 10, and 12 weeks. Error bars indicate ±SEM. (Mean and ±SEM values are represented in supplementary **table 8**). N=number of mice. The treatment groups are separated by vertical dashed lines. Statistical analysis was done by Two-way ANOVA followed by Bonferroni *post-hoc* test. Image brightness and contrast were adjusted for representative purpose. Full uncropped raw blots are represented in the supplementary figure S14.

Figure S7. Dynamics of mHTT aggregate formation across different stages of disease progression in R6/2.

A, B, C, D. Full uncropped raw western blot images for Fig. 1 showing mHTT aggregates in the region Cortex (C), Hippocampus (H), Striatum (S), and Cerebellum (CB) respectively from 2, 4, 8, and 12 weeks. The red dashed lines indicate the cropped area in the original blot in the main figure.

Figure S8. Basal autophagy is not altered across different stages of disease progression in R6/2.

A, B, C, and D. Full uncropped raw western blot for Fig. 2 for ATG proteins- p62/SQSTM1, LC3B, GABARAPL2 (GL2) from 2, 4, 8, and 12 weeks in the cortex, Hippocampus, striatum and cerebellum respectively. β-ACTIN is used as a loading control. The red dashed lines indicate the cropped area in the original blot in the main figure.

Figure S9. UBIQUITIN profile of the cell is unchanged despite the presence of mHTT aggregates across different stages of disease progression in R6/2.

A, B, C and D. Full uncropped raw western blot for Fig. S2 for UBIQUITIN profile from 2, 4, 8, and 12 weeks in the cortex, the Hippocampus, the striatum and the cerebellum, respectively**.** β-ACTIN is used as a loading control. The red dashed lines indicate the cropped area.

Figure S10. Nilotinib (Tasigna™) does not affect inducing autophagy in wild-type control mice.

A, B, C and D. Full uncropped raw western blot for Fig. 5 for ATG proteins- p62/SQSTM1, LC3B, GABARAPL2 (GL2) from 6, 8, 10 and 12 weeks in cortex, Hippocampus, striatum and cerebellum respectively in wild-type control mice. β-ACTIN is used as a loading control. The red dashed lines indicate the cropped area in the original blot in the main figure.

Figure S11. Nilotinib (Tasigna™) is ineffective in inducing autophagy across different stages of disease progression in R6/2.

A, B, C and D. Full uncropped raw western blot for Fig. 6 for ATG proteins- p62/SQSTM1, LC3B, GABARAPL2 (GL2) in cortex, hippocampus, striatum and cerebellum respectively from 6, 8, 10 and 12 weeks in R6/2 mice. β-ACTIN is used as a loading control. The red dashed lines indicate the cropped area in the original blot in the main figure.

Figure S12. Nilotinib (Tasigna™) is ineffective in enhancing the clearance of mHTT aggregates at any given stage of disease progression in R6/2.

A, B, C and D. Full uncropped raw western blot for Fig. 7 showing no effect of Tasigna in clearing mHTT aggregates (EM48) from 6, 8, 10, and 12 weeks in the cortex, hippocampus, striatum and cerebellum respectively in R6/2 mice. The red dashed lines indicate the cropped area in the original blot in the main figure.

Figure S13. Nilotinib (Tasigna™) has no effect on UBIQUITIN profile of the cell in wild-type control mice.

A, B, C and D. Full uncropped raw western blot for Fig. S5 for UBIQUITIN profile from 6, 8, 10, and 12 weeks in the cortex, Hippocampus, striatum and cerebellum in wild-type control mice, respectively. β-ACTIN is used as a loading control. The red dashed lines indicate the cropped area in the original blot in the main figure.

Figure S14. Nilotinib (Tasigna™) does not affect the UBIQUITIN profile of the cell across different stages of disease progression in R6/2.

A, B, C and D. Full uncropped raw western blot for Fig. S6 for UBIQUITIN profile from 6, 8, 10, and 12 weeks in the cortex, Hippocampus, striatum and cerebellum respectively in R6/2 mice. β-ACTIN is used as a loading control. The red dashed lines indicate the cropped area in the original blot in the main figure.

Figure S15. Nilotinib (Tasigna^{TM}) induces autophagy in HeLa cells in a dosage-dependent manner.

A. Full uncropped raw western blot for Fig. S3 for LC3B expression upon treatment with Nilotinib (Tasigna^{TM}). β-ACTIN is used as a loading control. The red dashed lines indicate the cropped area in the original blot in the main figure.
